# Genome homeostasis defects drive enlarged cells into senescence

**DOI:** 10.1016/j.molcel.2023.10.018

**Published:** 2023-11-16

**Authors:** Sandhya Manohar, Marianna E. Estrada, Federico Uliana, Karla Vuina, Patricia Moyano Alvarez, Robertus A.M. de Bruin, Gabriel E. Neurohr

**Affiliations:** 1 Institute for Biochemistry, Department of Biology, ETH Zürich 8093, Zürich, Zürich, Switzerland; 2 Laboratory for Molecular Cell Biology, University College London, London WC1E 6BT, UK; 3 UCL Cancer Institute, University College London, London WC1E 6BT, UK

**Keywords:** DNA damage, cell size, cell growth, cell cycle, senescence

## Abstract

Cellular senescence refers to an irreversible state of cell-cycle arrest and plays important roles in aging and cancer biology. Because senescence is associated with increased cell size, we used reversible cell-cycle arrests combined with growth rate modulation to study how excessive growth affects proliferation. We find that enlarged cells upregulate p21, which limits cell-cycle progression. Cells that re-enter the cell cycle encounter replication stress that is well tolerated in physiologically sized cells but causes severe DNA damage in enlarged cells, ultimately resulting in mitotic failure and permanent cell-cycle withdrawal. We demonstrate that enlarged cells fail to recruit 53BP1 and other non-homologous end joining (NHEJ) machinery to DNA damage sites and fail to robustly initiate DNA damage-dependent p53 signaling, rendering them highly sensitive to genotoxic stress. We propose that an impaired DNA damage response primes enlarged cells for persistent replication-acquired damage, ultimately leading to cell division failure and permanent cell-cycle exit.

## Highlights

• Excess G_1_ cell size triggers long-term cell-cycle exit via the p53-p21 pathway
• 53PB1 and other NHEJ machinery are not recruited to DNA breaks in enlarged cells
• This correlates with inefficient DNA damage repair and blunted p53 signaling
• Enlarged cells accumulate replication-acquired damage and undergo mitotic errors

## Graphical abstract

**Figure:**
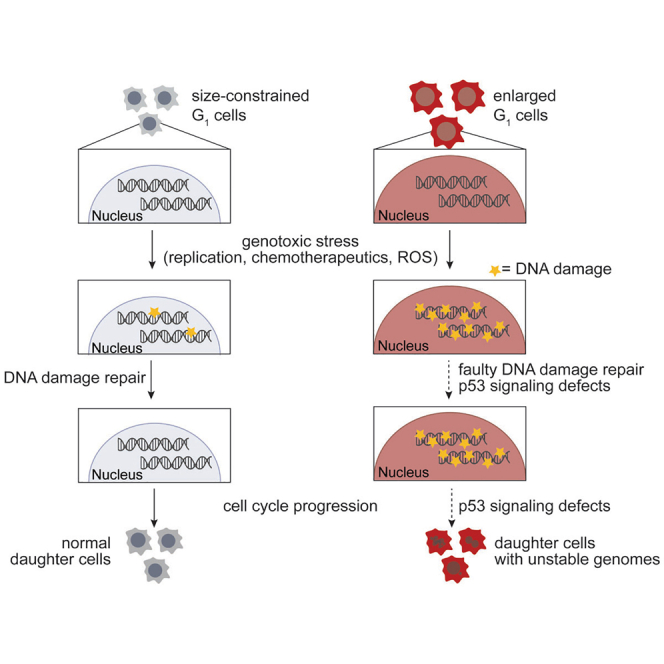

## Teaser

Manohar et al. studied how increased cell size—a hallmark of senescence—impairs long-term proliferation. Excess cell size activates p53-p21 signaling and impairs 53BP1 recruitment to DNA double-stranded breaks. This defect correlates with an impaired DNA damage response, DNA damage sensitivity, and genomic instability, resulting in permanent cell-cycle exit.

## Introduction

Cellular senescence describes a permanent state of cell-cycle arrest induced by diverse exogenous and endogenous stressors.^1^^,^^2^ Many cancer therapies trigger senescence and thus permanent cell-cycle withdrawal in tumor cells.^3^ Still, although senescence suppresses proliferation, tumors containing persistent senescent cells can be more invasive and are associated with worse outcomes.^3^^,^^4^ Thus, identifying how short-term cellular insults can cause a durable loss of proliferative potential is essential for understanding the benefits and limitations of senescence induction as a therapeutic strategy.

Despite being caused by diverse stimuli, it has long been observed that senescent cells are typically larger than cycling cells.^5^ More recent work has demonstrated that increased cell size is sufficient to withdraw cells from the cell cycle.^6^^,^^7^^,^^8^^,^^9^^,^^10^^,^^11^^,^^12^ Still, it is unclear what pathways drive cell-cycle exit in cells that exceed their normal size limit and how these pathways are activated. Answering these questions will provide key insight into how permanent cell-cycle arrest is conferred in senescent cells.

Senescence is typically conferred through the Rb pathway or the p53/p21 pathway.^13^ Rb is a cell-cycle inhibitor that binds E2F-family transcription factors to inhibit the transcription of G_1_/S cyclins and DNA replication factors,^14^ thereby blocking cell-cycle entry. The p53/p21 pathway also inhibits cell-cycle progression. p53 is a transcription factor that is activated upon diverse cellular stresses.^15^^,^^16^^,^^17^^,^^18^^,^^19^ p21—one of p53’s main transcriptional targets—is a Cdk1/2/4/6 inhibitor that halts cell-cycle progression.^20^ There is considerable cross talk between the p53/p21 and Rb pathways: because Rb mediated inhibition of E2F is dictated by Cdks, high levels of p21 block Cdk activity and therefore prevent E2F activation. Moreover, Rb directly regulates p53 stability through its interaction with MDM2, a p53-directed ubiquitin ligase.^21^ Still, it is unclear if increased cell size activates either or both of these pathways.

Here, we show that continued cell growth is required to induce long-term cell-cycle exit following a prolonged G_1_ cell-cycle arrest. We show that enlarged cells upregulate p21, which partially protects against cell-cycle entry and subsequent cell-cycle failure. Though a fraction of enlarged cells remains arrested in G_1_ following palbociclib removal, the remainder re-enters the cell cycle and acquires damage from replication that is not efficiently repaired, resulting in mitotic failures and cell-cycle exit. These cell-cycle progression abnormalities are accompanied by defective 53BP1 recruitment to DNA damage sites and a failure to robustly stimulate damage-dependent p53 signaling, rendering cells highly sensitive to genotoxic insults. We propose that these signaling defects prime cells for replication-acquired damage that is not repaired expediently, leading to mitotic failure followed by permanent cell-cycle withdrawal. Together, these results establish the consequences of excess size on cell-cycle progression and show that enlarged cells are prone to DNA damage, at least in part due to defects in DNA damage signaling and repair.

## Results

### Excess cell size induces permanent cell-cycle exit following a prolonged G_1_ arrest

**Figure 1:**
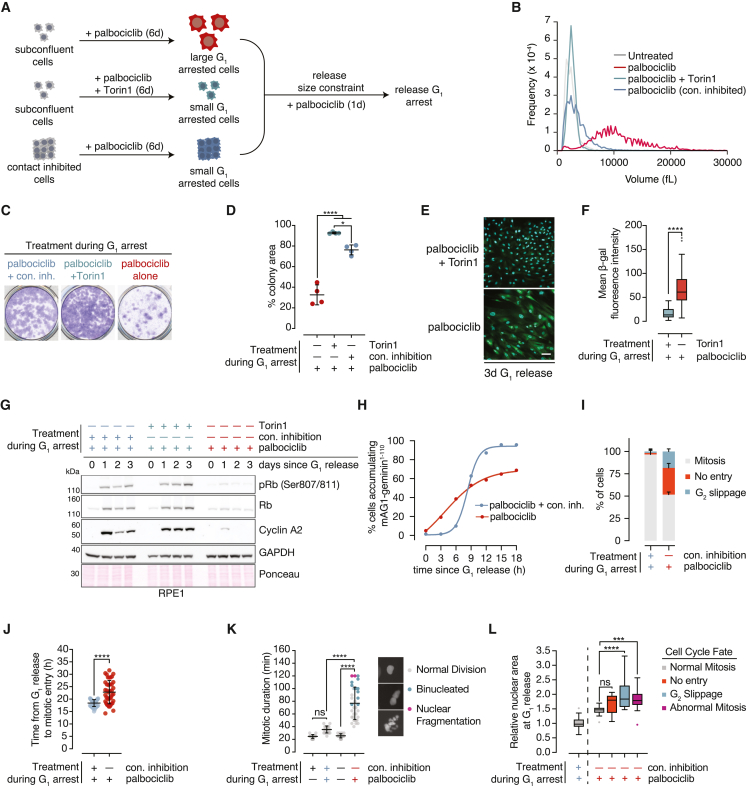
Excess cell size induces permanent cell-cycle exit following a prolonged G_1_ arrest (A) Schematic for altering G_1_ cell size. After 6 days of the indicated treatment, cells were re-seeded at sub-confluency and switched to media containing palbociclib alone for 24 h before conducting downstream experiments. (B) Coulter Counter-based cell volume measurements for RPE1 cells treated as in (A). (C) RPE1 cells were treated as in (A) and re-seeded in the absence of drugs for 10 days. Colonies were visualized with crystal violet. (D) Quantification of (C), n = 4. p values: one-way ANOVA followed by Tukey’s multiple comparisons test. (E) Representative images of enlarged and size-constrained (Torin1) RPE1 cells released from a G_1_ arrest and stained for SA-β-galactosidase. Scale bar, 100 μm. (F) Quantification of (E). >40 cells were analyzed per condition. Data are represented as Tukey plots. p value: two-tailed, unpaired t test. (G) RPE1 cells were treated as in (A) and western blot analysis of the indicated protein abundances was performed at the indicated timepoints following drug washout. GAPDH and Ponceau staining were used as loading controls. (H) RPE1 FUCCI cells were treated as in (A) using contact inhibition to constrain cell size and were imaged following release. The fraction of cells that accumulated mAG1-geminin^1–110^ above an arbitrary intensity threshold (indicating cell-cycle entry) is plotted at each time point. >45 cells were analyzed for each condition and time point. (I) RPE1 FUCCI cells were treated as in (H). Fates were scored for 20 randomly selected cells. n = 3 independent experiments (60 cells total for each condition). Error bars = mean + SD. (J) The timing from G_1_ release until mitotic entry for the first 40 cells that reached mitosis (nuclear envelope breakdown, NEB) for the experiments described in (H) and (I). p value: two-tailed unpaired t test. (K) Mitotic duration (the time from NEB to flattening or division) and mitotic fates for cells treated as in (H)–(J) that reached mitosis. p values (mitotic duration): one-way ANOVA followed by Tukey’s multiple comparisons test. (L) Correlation between nuclear area at G_1_ release and cell-cycle fate. Nuclear areas were normalized to the mean of size-constrained cells. “Abnormal mitosis” indicates that a given cell had an extended mitosis (>50 min) and/or a mitosis that culminated in nuclear abnormalities as shown in (K). >15 cells were quantified per fate from two experimental replicates. Data are represented as Tukey plots. p values: one-way ANOVA followed by Tukey’s multiple comparisons test.

To understand how increased cell size influences cell-cycle progression, we used palbociclib to arrest hTERT-RPE1 (hereafter referred to as RPE1) and MCF7 cells in G_1_ for an extended duration. Because G_1_-arrested cells continue to accumulate biomass,^7^ this treatment enables continued growth in the absence of division and significantly increases cell size. To disentangle the effects of increased size from those caused by prolonged Cdk4/6 inhibition, we employed two control strategies: (1) seeding cells at high confluence (contact inhibition) prior to palbociclib treatment and (2) co-inhibiting mTOR activity using the small molecule Torin1 (Figure 1A). Using these approaches, we obtained enlarged G_1_-arrested RPE1 (Figures 1B and S1A–S1C) and MCF7 cells (Figures S1D and S1E) and corresponding control cells that were close in size to untreated cells despite experiencing a G_1_ cell-cycle arrest for the same duration. Cells for which growth was restricted using either of these strategies are hereafter termed “size-constrained” (Figure 1A). In both cases, size-constrained cells were plated in palbociclib alone for 1 day following arrest to allow them to re-attach and recover from the effects of Torin1 treatment or contact inhibition. G_1_ arrest in RPE1 cells treated using this scheme was confirmed using FUCCI reporters^22^ (Figure S1A) and EdU incorporation (Figure S1B).

Cells that have grown beyond their physiological size range fail to proliferate and enter senescence.^6^^,^^7^^,^^23^^,^^24^ In agreement with these observations, we found that constraining cell size using contact inhibition or Torin1 treatment is sufficient to rescue long-term proliferation following release from a palbociclib-mediated G_1_ arrest (Figures 1C, 1D, S1F, and S1G). Enlarged RPE1 cells that were released from G_1_ also express high levels of senescence-associated β-galactosidase (SA-β-gal) (Figures 1E and 1F). Similarly, long-term proliferation defects in cells treated with a low dose of nutlin-3a are suppressed by constraining cell size during the arrest (Figures S1H and S1I). Thus, a prolonged cell-cycle arrest causes the long-term loss of proliferative potential and senescence induction, which can be rescued by limiting cell growth during the arrest.

To understand why G_1_ arrested enlarged cells undergo cell-cycle failure, we monitored cell-cycle markers following release in enlarged and size-constrained cells. We found that size-constrained cells recover and maintain cyclin A2 expression following release from G_1_ arrest (Figures 1G and S1J), indicating that they continue to cycle. In contrast, RPE1 cells that grew large recover a fraction of cyclin A2 expression only transiently (Figure 1G). Moreover, enlarged RPE1 cells display essentially no detectable phosphorylated Rb following a 3-day release—another sign of permanent cell-cycle exit (Figure 1G)^25^ These data suggest that at least a fraction of enlarged RPE1 cells enter the cell cycle once following G_1_ release before permanently exiting the cell cycle.

To further understand the events that lead to cell-cycle failure in enlarged RPE1 cells, we used live-cell imaging to visualize cell-cycle re-entry following G_1_ release. Using the scheme shown in Figure 1A, we monitored cell-cycle progression in enlarged and size-constrained RPE1 cells expressing FUCCI cell-cycle reporters^22^ (Figure S1K; Videos S1, S2, and S4). Although essentially all size-constrained cells re-enter the cell cycle, approximately 30% of enlarged cells remain arrested in G_1_ (Figures 1H and 1I). Of the remaining enlarged cells that re-enter the cell cycle, the majority progress to mitosis, whereas the remainder re-enter G_1_ without going through mitosis (“G_2_ slippage”). In contrast, essentially all size-constrained cells progress to mitosis normally (Figure 1I).

**Figure 2:**
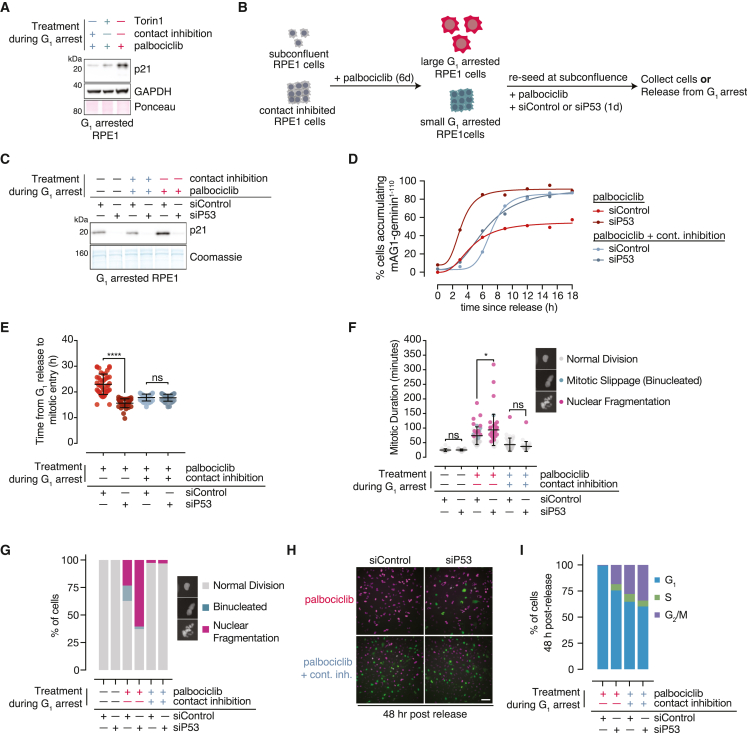
Excess G_1_ cell size activates p53-dependent signaling in RPE1 cells (A) Western blot of p21 protein levels in size-constrained and enlarged G_1_-arrested RPE1 cells. GAPDH and Ponceau staining are loading controls. (B) Schematic for p53 knockdown experiments. (C) p21 protein levels in cells treated as in (B). Coomassie staining of the gel was used as a loading control. (D–I) RPE1 FUCCI cells were treated as in (B) and were imaged for 48 h after drug washout. (D) Cell-cycle entry was calculated based on the number of cells that surpassed an arbitrary mAG1-geminin^1–110^ threshold following washout. >90 cells were analyzed for each condition and time point. (E) Time from G_1_ release until mitotic entry for the first 40 cells that reach mitosis in (D). p values: one-way ANOVA followed by Tukey’s multiple comparisons test. (F) Quantification of mitotic duration and mitotic failure in cells that reached mitosis +/− p53 knockdown. >25 cells were quantified per condition. p values (mitotic duration): two-way ANOVA followed by Tukey’s multiple comparisons test. Error bars = mean ± SD. (G) Mitotic errors indicated in (F) shown as a fraction of total cells that entered mitosis. (H) Representative images 48 h after G_1_ arrest release. Magenta indicates mCherry-Cdt1^30–120^, whereas green indicates mAG1-geminin^1–110^. Scale bar, 100 μm. (I) Quantification of (H). >3 images were analyzed per condition with 80–200 cells per image.

Video S1. Cycling RPE1 FUCCI cells, related to Figures 1 and 2

Video S2. Size-constrained RPE1 FUCCI cells released from G_1_ (siControl transfected), related to Figures 1 and 2

In the fraction of enlarged cells that reach mitosis, mitotic entry is delayed relative to size-constrained cells (Figure 1J). Large cells also spend significantly longer in mitosis and have a high frequency of abnormal mitotic outcomes, including nuclear fragmentation and mitotic slippage yielding binucleated cells (Figure 1K). We observed similar post-mitotic defects in fixed RPE1 WT cells using nuclear staining following release (Figure S1L), confirming that these phenotypes are not artifacts from long-term imaging.

Given the heterogeneous cell size distribution of enlarged cells (Figure 1B), we examined cell-cycle progression defects as a function of cell size within the enlarged cell population. We found that the median nuclear area (a proxy for cell size^26^^,^^27^) at G_1_ release of cells that go on to experience cell-cycle progression defects is significantly greater than that of cells that undergo normal cell-cycle progression (Figure 1L). Interestingly, we found a wide distribution of nuclear areas for enlarged cells that fail to re-enter the cell cycle at all (“no entry”), suggesting that additional factors may drive the decision to remain in G_1_ (Figure 1L).

To assess whether the effects of prolonged cell-cycle arrest on cell size and proliferation that we observed in RPE1 and MCF7 cells are shared in other cell lines, we also studied the B-cell precursor leukemia cell line NALM6, which also arrests in G_1_ following palbociclib treatment (Figure S1M). Interestingly, we observed that G_1_-arrested NALM6 cells fail to accumulate biomass to the same extent as RPE1 and MCF7 cells (Figure S1N). Consistent with our hypothesis that cell size is the determinant of cell-cycle progression following G_1_ arrest release, NALM6 cells display neither cell-cycle entry nor long-term proliferation defects following release from a 6-day palbociclib arrest (Figures S1O and S1P). Thus, preventing excess biomass accumulation during a prolonged G_1_ arrest preserves long-term proliferative potential.

Together, our data indicate that excess cell size causes cell-cycle progression abnormalities and a propensity for prolonged, erroneous mitoses. The specific defects we observed in enlarged cells that re-enter the cell cycle (G_2_ slippage and mitotic failures) are consistent with unresolved, replication-acquired DNA damage.^28^ Because we observe these phenotypes only in enlarged cells, we conclude that the cell-cycle failure and senescence induction observed following palbociclib treatment are a consequence of increased cell size and are not merely due to a prolonged G_1_ cell-cycle arrest.

### Excess G_1_ cell size activates p53-dependent signaling in RPE1 cells

Enlarged G_1_-arrested cells express high levels of the Cdk inhibitor p21 (CDKN1A) relative to size-constrained cells (Figures 2A and S2A). Using siRNA-mediated knockdown of p53, we found that the upregulation of p21 in enlarged G_1_ RPE1 cells is p53 dependent (Figures 2B and 2C). This does not require active ATM or ATR signaling (Figures S2B–S2E) and is therefore not initially triggered by activation of the canonical DNA damage response.^29^

Because p21 is a Cdk inhibitor,^20^^,^^30^ we examined whether p53 depletion affects cell-cycle entry in enlarged cells (Figure S2F; Videos S2, S3, S4, and S5). Indeed, p53 depletion eliminates the fraction of enlarged cells that fail to enter the cell cycle, hastens the G_1_/S transition (Figure 2D), and eliminates the mitotic entry delays we observed in enlarged cells (Figure 2E). In agreement with this finding, related work shows that p21 depletion promotes cell-cycle entry in enlarged cells.^31^ p53 depletion in enlarged cells also increases the frequency of mitotic failures but has no effect on cell-cycle progression in size-constrained cells (Figures 2F and 2G). This result indicates that p53 protects against catastrophic cell division failure in enlarged RPE1 cells but is dispensable for at least one cell cycle in size-constrained cells. Together, these data indicate that cell-cycle progression in enlarged RPE1 cells is restrained by active p53 signaling.

Video S3. Size-constrained RPE1 FUCCI cells released from G_1_ (siP53 transfected), related to Figure 2

Video S4. Enlarged RPE1 FUCCI cells released from G_1_ (siControl transfected), related to Figures 1 and 2

Video S5. Enlarged RPE1 FUCCI cells released from G_1_ (siP53 transfected), related to Figure 2

p21 is also upregulated in enlarged MCF7 cells but—in contrast to our findings in RPE1 cells—this is not affected by p53 knockdown (Figure S2G). This could be a consequence of incomplete p53 depletion using an siRNA. We also ruled out the possibility that p21 levels in enlarged MCF7 cells are driven by p73 (Figure S2H). Similar to enlarged RPE1 cells, a significant fraction of enlarged MCF7 cells fail to re-enter the cell cycle following G_1_ release (Figures S2I and S2J). Whereas p53 knockdown had no effect on the fraction of enlarged MCF7 cells that re-enter the cell cycle following G_1_ release, p21 knockdown rescued cell-cycle entry to the level of size-constrained cells (Figures S2I and S2J). In addition, we observed an increase in nuclear abnormalities in enlarged cells that re-enter the cell cycle following p21 knockdown (Figures S2K and S2L). These data, combined with our observations in RPE1 cells, demonstrate that p21 is the critical factor for restraining cell-cycle progression following G_1_ release in two different enlarged cell lines.

Following one round of cell division, enlarged RPE1 cells almost all arrest in G_1_, likely reflecting irreparable DNA damage accrued during the previous cell cycle (Figures 2H and 2I). In contrast and consistent with previous experiments, siP53-transfected large cells continue to cycle.^10^^,^^32^ This occurs even in cells that have undergone mitotic catastrophes (Figure 2H). Thus, p53-dependent p21 expression limits cell-cycle entry and mitotic failure in enlarged RPE1 cells and also prevents subsequent cell-cycle re-entry following an initial cell-cycle failure, thereby limiting the propagation of unstable genomes.

### Enlarged cells acquire high levels of DNA damage from endogenous replication stress

**Figure 3:**
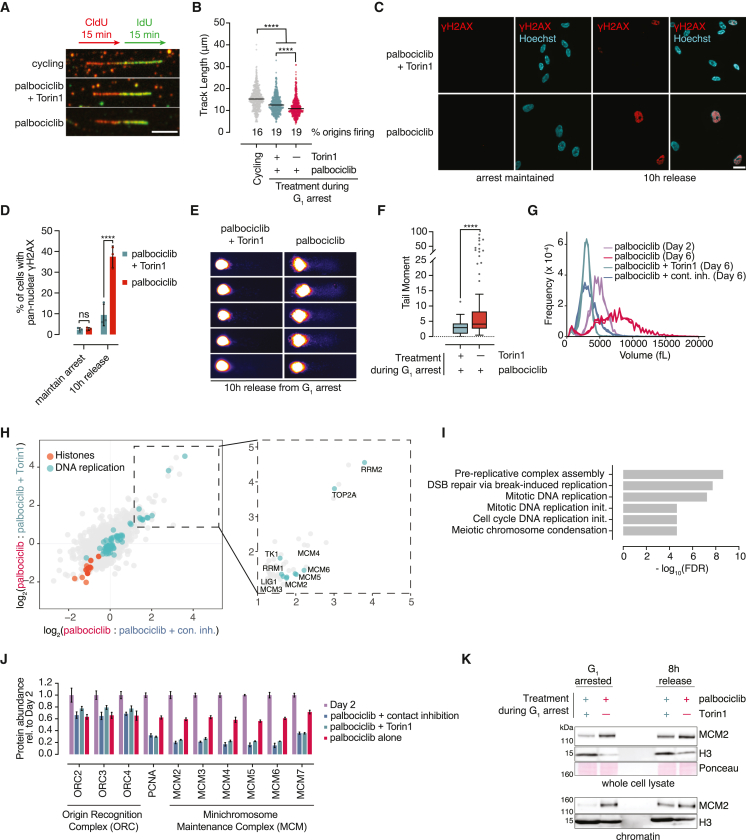
DNA replication machinery is not limiting in enlarged G_1_ cells (A) Representative DNA fibers for enlarged and size-constrained RPE1 cells that were released from G_1_ for 14 h. Scale bar, 5 μm. (B) Quantification of (A). >480 fibers from three independent experiments were scored. Bars represent the median track length. p values: Kruskal-Wallis test (one-way ANOVA on ranks). (C) Representative images of γH2AX immunostaining for siP53 transfected RPE1 cells that were either maintained in a G_1_ arrest (left) or released for 10 h (right). Scale bar, 25 μm. (D) Quantification of (C). Individual data points represent three independent experiments, with 50–100 cells analyzed each. p values: two-way ANOVA followed by Tukey’s multiple comparison test. Error bars = mean ± SD. (E) Representative images of alkaline comets derived from cells treated as in (C). (F) Quantification of (E). The tail moment (see STAR Methods section) of >120 comets from two replicates were scored for each condition. Data are represented as Tukey plots. p value: two-tailed, unpaired t test. (G) Cell volume measurements for TMT-based MS experiment (H–J). (H) Comparison of relative protein abundances in enlarged cells vs. either Torin1 (y axis) or contact inhibited (x axis) cells. (Inset) Upregulated proteins in palbociclib-treated cells with a log_2_(fold change) ≥ 1. (I) Gene ontology (GO) for biological processes for proteins in the inset of (H). (J) MS-acquired relative protein abundances of selected replisome components. Values are normalized to the mean of the day 2 value. Error bars: mean ± SD. (K) Western blots of whole-cell lysate (top) and chromatin fractions (bottom) from G_1_-arrested (left) and 8 h released (right) cells. Ponceau staining is a loading control for whole-cell lysates. H3 is a loading control for the chromatin fractions.

The mitotic failures and G_2_ slippage observed in enlarged cells (Figures 1I, 1K, S1L, 2F, 2G, S2K, and S2L) are hallmarks of unrepaired, replication-acquired damage at mitotic entry.^33^^,^^34^^,^^35^ Based on these observations, we investigated whether enlarged cells undergo replication stress that could explain subsequent cell-cycle failures. Using a DNA fiber assay, we and others^31^ found that G_1_-released enlarged cells display a modest decrease in replicative track length compared with cycling cells, indicating replication fork slowdown. This is partially rescued by constraining cell size (Figures 3A, 3B, and S3A). Thus, both enlarged and size-constrained cells released from a G_1_ arrest encounter mild replication stress. Although it is yet unclear what causes this replication stress, our data are not consistent with under-licensing of replication origins—which has been previously proposed in palbociclib-treated cells^10^—because we do not observe reduced origin firing (Figures 3B and S3A).

Our finding that constraining cell size only partially suppresses replication stress was surprising given that only enlarged cells display cell-cycle progression defects indicative of unrepaired damage. We therefore hypothesized that the replication stress we observed in our DNA fiber analysis is sufficient to cause high levels of damage in enlarged cells but not in size-constrained cells. Consistent with this hypothesis, we detected more DNA damage in enlarged RPE1 cells that had progressed to G_2_ using γH2AX staining^36^ and an alkaline comet assay (Figures 3C–3F). These experiments were performed in a p53 knockdown background to increase the synchrony between enlarged and size-constrained cells (Figure 2E). Together, these results indicate that mild replication stress correlates with high levels of DNA damage in enlarged cells but has little effect on size-constrained cells.

Based on the difference we observe in DNA damage levels in enlarged vs. size-constrained cells (Figures 3C–3F), we reasoned that enlarged cells have additional defects that sensitize them to replication aberrations. Previous studies have reported that palbociclib-treated cells downregulate origin licensing components over time.^10^ Although replication origins are licensed in excess of what is required for a normal S-phase, this surplus of licensed origins becomes essential for cell-cycle progression during replication stress.^37^^,^^38^ We thus hypothesized that changes in the abundance of origin licensing or other DNA replication factors could explain the increased sensitivity to replication stress observed in enlarged cells.

To address this hypothesis, we used tandem mass tag (TMT)-based quantitative proteomics to compare relative protein abundances in enlarged and size-constrained cells. To identify proteins that change in response to G_1_ arrest duration as opposed to size, we also included a 2-day palbociclib-arrest time point (Figure 3G). Using this setup, we identified and quantified 5,884 proteins based on at least two peptides (Figures 3H and S3B–S3G; Table S1). Of note, Lamin B1 and HMGB2 were depleted in enlarged cells, which are known features of senescent cells (Figure S3H).^39^^,^^40^^,^^41^^,^^42^

To identify proteins whose abundances are differentially regulated in large cells relative to both of the size-constrained conditions we used, we compared protein abundances in large cells with each size-constrained condition (Figure 3H, left). We identified 44 proteins whose abundances decreased (log_2_FC < −1, adj. p value < 0.05) in enlarged cells relative to both size-constrained conditions (Table S2). This subset of proteins was comprised mostly of histones (Figure 3H), which was expected because histones scale with DNA content rather than cell size.^11^^,^^43^ We also identified 50 proteins whose abundances increased (log_2_FC > 1, adj. p value < 0.05) in enlarged cells relative to both size-constrained conditions (Table S3; Figure 3H, inset). Gene ontology (GO) analysis of this subset revealed an enrichment in DNA replication and cell-cycle proteins (Figures 3I and S3I). Thus, many factors that are important for S-phase are actually more abundant in enlarged cells (Figure 3H, inset). Together, these data indicate that DNA replication protein depletion is unlikely to explain the replication stress sensitivity observed in enlarged cells.

Regarding the abundances of origin licensing factors, our 2-day and 6-day palbociclib-arrested cells reproduce previous findings^10^: we observe a loss of MCM complex components, ORC components, and PCNA as a function of G_1_ arrest duration (Figure 3J). Still, we found that ORC components are equally abundant between enlarged and size-constrained cells (Figure 3J). Moreover, PCNA and MCM components are more abundant in enlarged cells. Consistent with this, we found that G_1_ arrested enlarged cells have more chromatin-associated MCM than size-constrained cells and that this difference is eliminated after an 8 h release (Figure 3K). These data suggest that replisome abundance and loading are not limiting in enlarged cells. Therefore, origin licensing deficits cannot explain the replication stress sensitivity we observe in enlarged cells.

Our data demonstrate that both size-constrained and enlarged cells are subject to mild DNA replication stress, but only enlarged cells accumulate DNA damage. Because this could not be explained by the altered expression of DNA replication factors, we next investigated whether enlarged cells may harbor a general sensitivity to genotoxic stress.

### Excess cell size dampens DNA damage-induced p53 signaling

**Figure 4:**
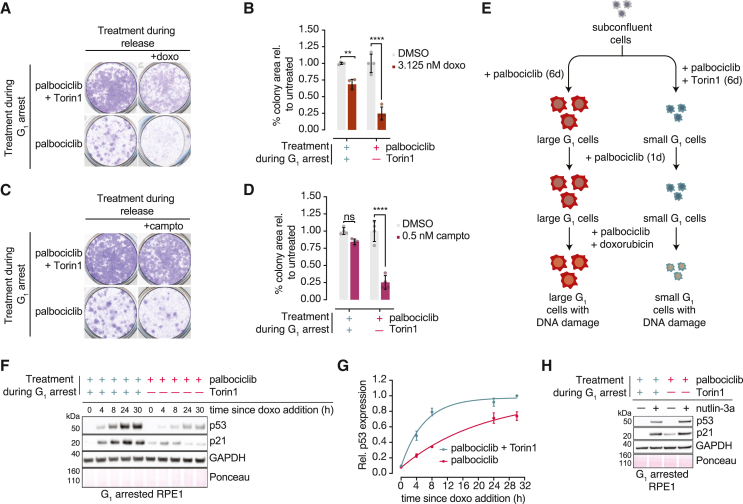
Excess cell size dampens DNA damage-induced p53 signaling (A) RPE1 cells were treated as in Figure 1A and re-seeded in media containing DMSO or 3.125 nM doxorubicin. Colonies were visualized with crystal violet after 10 days. (B) Quantification of (A). n = 4. p values: two-way ANOVA followed by Tukey’s multiple comparison test. Values were normalized to the DMSO condition. Error bars: mean ± SD. (C) Cells were treated as in (A) but with 0.5 nM camptothecin instead of doxorubicin. (D) Quantification of (C). n = 4. Statistical analysis was carried out as in (B). (E) Scheme for treating G_1_-arrested cells with doxorubicin. Doxorubicin treatment was carried out in the continuous presence of palbociclib for an additional 24 h. (F) RPE1 cells were treated as in (E). Following doxorubicin addition, cells were collected at the indicated time points and lysates were analyzed by western blot. GAPDH and Ponceau staining are loading controls. (G) Quantification of p53 levels in (F). Protein levels were normalized to GAPDH and the 30 h size-constrained condition. n = 2, Error bars: mean ± range. (H) RPE1 cells were treated as in (E) with 5 μM nutlin-3a instead of doxorubicin. Protein abundances were measured by western blot. GAPDH and Ponceau staining are loading controls.

To understand whether excess cell size renders cells generally more sensitive to genotoxic stress, we asked whether enlarged cells are more sensitive to exogenous DNA damaging agents. We found that the proliferation of enlarged cells released into low doses of known DNA damaging agents is stunted compared with size-constrained cells (Figures 4A–4D and S4A–S4D). These data indicate that—in addition to replication—enlarged cells are broadly sensitive to genotoxic insults when released into the cell cycle. To disentangle the effects of cell-cycle progression from the response to DNA damage, we next analyzed how differently sized cells respond to DNA damage while still arrested in G_1_. This allowed us to better control for the level of damage exposure, because G_1_-arrested enlarged and size-constrained cells both have essentially no baseline damage (Figures 3C–3F).

p53 signaling is involved in the DNA damage response and is important for suppressing DNA damage-associated mitotic defects.^44^^,^^45^^,^^46^ We hypothesized that p53 signaling defects could potentially explain the high rate of mitotic failure we observed in enlarged cells that undergo replication (Figure 1K). Using the experimental scheme in Figure 4E, we found that enlarged G_1_ arrested, doxorubicin-treated cells accumulate less p53 relative to size-constrained cells (Figures 4F, 4G, and S4E). Moreover, p21 induction is blunted in doxorubicin-treated enlarged RPE1 cells (Figure 4F). Enlarged cells eliminate p53 with similar kinetics as size-constrained cells upon doxorubicin washout (Figures S4F and S4G), suggesting that p53 signaling can be silenced to the same extent. In contrast, we found that size-constrained and enlarged RPE1 cells treated with nutlin-3a—which stabilizes p53 without causing DNA damage—stabilize p53 to the same extent (Figure 4H). This suggests that large cells’ failure to mount a robust p53 response does not arise from defective p53 synthesis. Thus, we hypothesize that the p53 defects we observe in enlarged cells may be DNA damage-specific and could result from inadequate signaling.

### Enlarged G_1_ cells are prone to DNA damage

**Figure 5:**
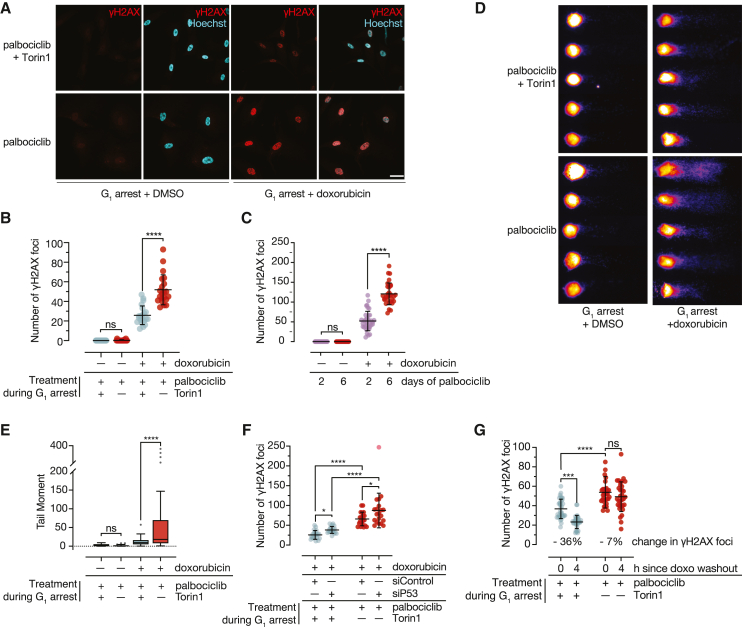
Enlarged G_1_ cells are prone to DNA damage (A) Cells were treated as in Figure 4E and immunostained for γH2AX. Scale bar, 40 μm. (B) Quantification of (A). >20 cells were quantified per condition. p values: one-way ANOVA followed by Tukey’s multiple comparisons test. Error bars: mean ± SD. (C) Cells that were arrested in palbociclib for 2 or 6 days were treated +/− doxorubicin for 1 day followed by immunostaining for γH2AX. Statistical analysis was carried out as in (B). (D) Cells were treated as in (A) but, instead, were subjected to an alkaline comet assay. Representative comets are shown. (E) Tail moment quantification of comets from the experiment shown in (D) as in Figure 3F. (F) Cells were treated as indicated and immunostained for γH2AX. Quantification was carried out as in (B) and (C). (G) Quantification of γH2AX staining in cells treated with 500 nM doxorubicin for 16 h (time = 0) and 4 h after doxorubicin washout (maintaining palbociclib). γH2AX foci were quantified as above.

Although blunted p53 signaling may explain why enlarged cells undergo cell-cycle progression in the presence of replication-acquired damage, it is unlikely to explain why replication stress causes high levels of DNA damage in enlarged cells but not in size-constrained cells. To understand whether enlarged cells’ propensity for DNA damage following replication is shared with other genotoxic stresses, we measured DNA damage levels following doxorubicin treatment using the scheme shown in Figure 4E. Although neither enlarged nor size-constrained G_1_ arrested RPE1 cells show basal signs of DNA damage, we found that enlarged cells harbor significantly more γH2AX foci than size-constrained G_1_ cells following doxorubicin treatment (Figures 5A–5C and S5A–S5D). Because H2AX is a histone and scales relative to genome content^11^^,^^43^ (Figure 3H), loading was normalized to cell number for western-blot-based γH2AX measurements (Figures S5B, S5C, and S5E).

We also measured DNA damage directly using an alkaline comet assay. Mirroring our γH2AX results, we found that untreated enlarged and size-constrained cells show no difference in basal DNA damage, whereas doxorubicin treatment causes significantly more damage in enlarged cells (Figures 5D and 5E). Because these experiments were carried out during a sustained G_1_ arrest, these data indicate that the increased propensity for DNA damage that we observed in enlarged cells following replication stress is already established during G_1_.

Because p53 contributes to DNA damage repair,^13^ we asked whether the p53 defects we observed in enlarged cells could account for the increased level of damage we observed in enlarged cells upon doxorubicin treatment. We found that p53 knockdown is sufficient to slightly increase the level of damage observed in both size-constrained and enlarged cells treated with doxorubicin (Figures 5F and S5E). Still, enlarged cells wherein p53 is knocked down still harbor higher levels of damage than the corresponding size-constrained cells (Figures 5F and S5E), indicating that—although p53 signaling defects may contribute to the high levels of DNA damage observed in enlarged cells—other factors must be involved.

To investigate whether the high levels of damage we observe in doxorubicin-treated enlarged cells could be due to DNA damage repair defects, we treated enlarged and size-constrained cells with doxorubicin for 16 h followed by a 4 h washout and measured γH2AX foci. We found that size-constrained cells eliminate γH2AX foci by 36% following doxorubicin removal, whereas enlarged cells only show a 7% reduction (Figure 5G). Thus, enlarged cells fail to clear DNA damage as efficiently as size-constrained cells, indicating defective DNA damage repair.

### Enlarged G_1_ cells display defects in the canonical NHEJ pathway

The γH2AX signal we observed in doxorubicin-treated G_1_-arrested enlarged and size-constrained cells is ATM dependent (Figure S6A), indicating that doxorubicin predominantly causes double-stranded breaks (DSBs) in G_1_-arrested cells.^47^ During G_1_, DSBs are mitigated by non-homologous end joining (NHEJ). Because enlarged G_1_ cells acquire more DNA damage upon DSB exposure (Figures 5A–5F) and fail to clear it to the same extent (Figure 5G) relative to size-constrained cells, we hypothesized that enlarged cells may harbor NHEJ defects. Moreover, because NHEJ components localize to stalled replication forks,^48^^,^^49^^,^^50^^,^^51^ defects in this pathway could also explain the failure to resolve replication stress in enlarged cells (Figures 3C–3F).

**Figure 6:**
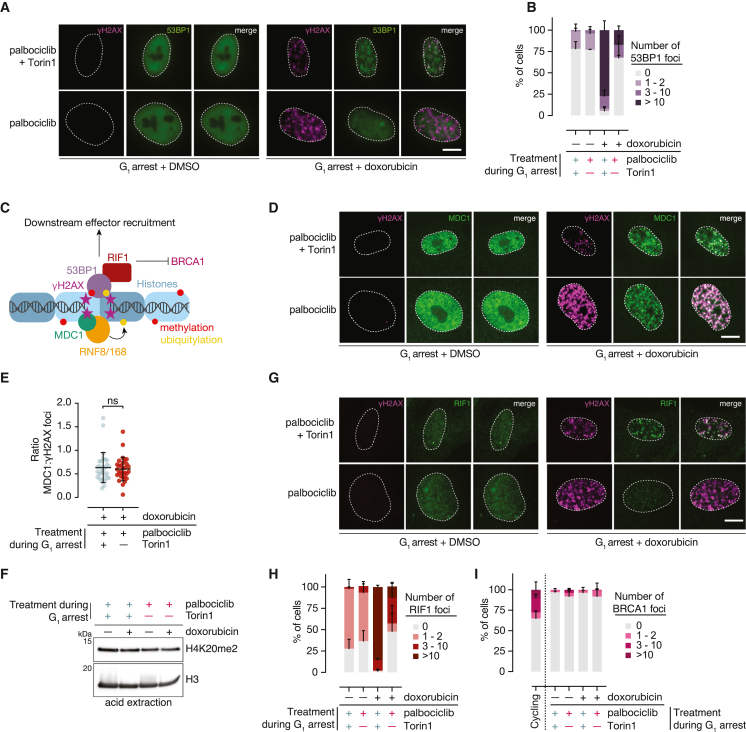
NHEJ signaling is compromised in enlarged G_1_ cells (A) G_1_ arrested RPE1 cells were treated as in Figure 4E and co-immunostained for 53BP1 and γH2AX. Scale bar, 10 μm. (B) Quantification of 53BP1 foci in (A). Data represent the mean of three independent experiments with at least 30 cells measured in each experiment. Error bars: mean + SD. (C) Key components of the non-homologous end joining (NHEJ) pathway. (D) Cells were treated as in (A) and co-stained for MDC1 and γH2AX. Scale bar, 10 μm. (E) Quantification of MDC1 and γH2AX foci in (D). MDC1 and γH2AX foci are plotted as a ratio 0 for each cell to quantify MDC1 recruitment. (F) Cells were treated as in (A). Equal amounts of acid-extracted histones were analyzed by western blotting. H3 is a loading control. (G) Cells were treated as in (A) and co-stained for RIF1 and γH2AX. Scale bar, 10 μm. (H) Quantification of RIF1 foci in (G). Data represent the mean of three independent experiments with at least 40 cells measured in each experiment. Error bars: mean + SD. (I) Cells were treated as in (A) and immunostained for BRCA1. Cycling cells were included as a control because BRCA1 foci form during a normal S-phase. Data represent the mean of three independent experiments. Error bars: mean + SD.

NHEJ involves the formation of discrete 53BP1 foci at damage sites.^52^ These foci then act as adaptors for downstream signaling and antagonize homology-directed repair (HR).^53^ To probe the NHEJ pathway in enlarged and size-constrained G_1_ cells, we measured 53BP1 foci formation upon doxorubicin treatment. We found that over half of doxorubicin-treated enlarged cells fail to form any 53BP1 foci, whereas size-constrained cells do so proficiently (Figures 6A and 6B). Thus, an upstream component that is essential for NHEJ is impaired in enlarged cells. This finding may also explain our observation that enlarged cells fail to robustly stabilize p53 in response to damage because others have shown that blocking 53BP1 foci formation blunts p53 induction.^52^^,^^54^^,^^55^^,^^56^

To investigate whether the failure of upstream steps in NHEJ initiation could explain large cells’ failure to deposit 53BP1 at damage sites, we systematically analyzed the NHEJ induction cascade (Figure 6C). 53BP1 foci formation depends on several upstream steps. First, MDC1 is recruited to damage sites.^53^^,^^57^ Next, MDC1 recruits RNF8, which subsequently recruits RNF168.^58^^,^^59^ RNF168 ubiquitylates histones proximal to damage sites which—along with H4K20 dimethylation marks^60^^,^^61^—are required for 53BP1 foci deposition.^58^^,^^59^

We first investigated MDC1 recruitment to damage sites. We found that G_1_ arrested enlarged cells treated with doxorubicin successfully recruit MDC1 to damage sites (Figure 6D). We measured more MDC1 foci in doxorubicin-treated enlarged cells than in size-constrained cells which—consistent with our γH2AX staining and comet results—reflects more damage (Figure S6B). When we compare the ratio of MDC1 foci with γH2AX foci in a given cell as a proxy for MDC1 recruitment per damage site, we find that enlarged and size-constrained cells are equally proficient at recruiting MDC1 (Figure 6E). Thus, MDC1 recruitment defects cannot explain enlarged cells’ failure to form 53BP1 foci.

Next, we investigated whether RNF168-dependent histone ubiquitylation is altered in enlarged cells. We found that there is a greater fraction of RNF168-dependent nuclear ubiquitin in enlarged cells, again reflecting higher levels of damage (Figures S6C and S6D). This suggests that RNF168-mediated histone ubiquitylation is intact and is unlikely to explain the 53BP1 foci recruitment defect we observe in enlarged cells.

Because 53BP1 recruitment also requires H4K20 dimethylation,^60^^,^^61^ we also measured H4K20me2. We found that enlarged and size-constrained cells have similar levels of H4K20me2 regardless of doxorubicin treatment (Figure 6F). Thus, we find no evidence of deficient H4K20 dimethylation in enlarged cells. Based on our analysis of the upstream regulators of 53BP1, we find no evidence that defects in the recruitment of these factors explain enlarged cells’ failure to accumulate 53BP1 at damage sites. Thus, the 53BP1 recruitment defect we observe in enlarged cells likely occurs at the level of 53BP1.

To determine whether the failure to recruit 53BP1 to damage sites impacts downstream signaling, we analyzed RIF1, which is directly recruited to damage sites by 53BP1. Together, 53BP1 and RIF1 block recruitment of the HR factor BRCA1 and recruit further downstream effectors (Figure 6C). Strikingly, we found that the loss of 53BP1 at damage sites in enlarged cells correlates with a failure to recruit RIF1 (Figures 6G and 6H). Because RIF1 is required for 53BP1-dependent NHEJ,^62^ this result demonstrates that enlarged cells’ failure to form 53BP1 foci impedes downstream signaling. Interestingly, the 53BP1 and RIF1 defects we observed did not correlate with aberrant BRCA1 recruitment in enlarged cells (Figures 6I and S6E), suggesting that there are other factors preventing BRCA1 recruitment in G_1_ arrested cells. In summary, excess cell size causes a failure to properly recruit NHEJ factors in response to DNA damage. Because 53BP1 and RIF1 are also recruited to stalled replication forks,^48^^,^^50^^,^^51^ these defects may also explain enlarged cells’ failure to mitigate replication stress and the resulting mitotic defects that ultimately cause permanent cell-cycle exit.

## Discussion

Here we show that cells that are released from a prolonged G_1_ arrest are subject to mild replication stress. Although the replication stress we observe in enlarged cells is partially rescued by constraining cell size, it is not clear whether this is sufficient to explain why only enlarged cells obtain high levels of DNA damage. Our proteomic data show that this discrepancy is unlikely to be due to deficiencies in proteins that are important for cell-cycle progression following replication stress. Indeed, many replication-associated proteins are actually more abundant in enlarged cells. This observation is likely explained by the fact that both of the size-constraint methods employed in this experiment suppress mTOR signaling,^63^ which is known to repress the expression of E2F targets, many of which are involved in DNA replication.^64^^,^^65^ Still, because replication proteins are not limiting, there are two remaining possible explanations for the high levels of damage observed in enlarged cells: (1) enlarged cells’ DNA is inherently breakage-prone, and (2) compromised repair pathways prevent damage mitigation. This work does not address the first possibility, which is an intriguing topic for future research. Instead, we confirm the second possibility and show that excess G_1_ cell size impairs the DNA damage response, leading to high levels of DNA damage following genotoxic stress exposure in enlarged cells.

We find that enlarged cells fail to form 53BP1 foci in response to DSBs, demonstrating a critical failure in the NHEJ pathway. The 53BP1 defects we observe in enlarged cells are consistent with previous observations: aged G_1_ cells—which are larger than healthy cells^66^—also fail to recruit 53BP1 to DSB sites.^67^ Others have shown that 53BP1 foci formation is highly dependent on the biophysical properties of the nucleoplasm.^54^ Reduced macromolecular crowding resulting from nuclear dilution has been previously described in senescent cells^68^^,^^69^ and could impair 53BP1’s ability to undergo liquid-liquid phase separation.^54^^,^^56^^,^^70^ Similarly, although we observe no defects in overall H4K20me2 abundance, it is possible that nuclear dilution and/or differences in chromatin architecture change the accessibility of this mark, precluding 53BP1 foci formation.

Our results present important clinical implications for the mechanism by which Cdk4/6 inhibitors induce permanent cell-cycle withdrawal in cancer cells. Palbociclib and other Cdk4/6 inhibitors are used clinically for the treatment for HR+ and HER2- breast cancers.^71^ Although others have shown that prolonged Cdk4/6 inhibition causes cell-cycle failure,^10^^,^^31^^,^^32^^,^^72^^,^^73^ our data demonstrate that this is cell size dependent. This distinction suggests that Cdk4/6 inhibition may be a more useful therapeutic strategy for tumors containing cells that are susceptible to unchecked biomass accumulation. Consistent with this idea, others have shown that oncogenic mutations that amplify cell growth sensitize cells to palbociclib treatment,^73^ and hyperactivation of mTOR (which also amplifies cell growth) sensitizes estrogen receptor positive breast cancer cells to Cdk4/6 inhibition in terms of permanent cell-cycle withdrawal.^74^ Thus, this work identifies potential guidelines for the efficacy of Cdk4/6 inhibitors in a therapeutic context.

### Limitations of the study

The study presented here identifies specific features of the DNA damage repair system that are compromised as a function of excess cell size. Although we have identified a specific defect in the NHEJ pathway, further work is still required to understand how excess cell size causes this defect. Although our findings give new insight into why excess cell size causes long-term proliferative failure, a caveat is that the experiments described here were conducted in 2D cell culture using immortalized cell lines. Although this enabled us to conduct mechanistic studies in a manner that more complex tissues would not have allowed, it is not yet clear whether primary cells within a complex tissue experience excess growth to the same extent as cultured cells upon palbociclib treatment. Although senescent cells become enlarged *in vivo*,^75^ additional work should be conducted to understand how palbociclib affects cell size within tissues. Additionally, because much of our data uses contact inhibition, mTOR inhibition, and arrest duration modulation to constrain cell size, our results should be interpreted with potential pleiotropic effects in mind.

## STAR★Methods

### Key resources table

**Table:** 

REAGENT or RESOURCE	SOURCE	IDENTIFIER
**Antibodies**		

Rb	Cell Signaling Technology	#9309
pRb (Ser807/811)	Cell Signaling Technology	#9308
cyclin A2	Santa Cruz	sc-751
GAPDH	Abcam	ab8245
Vinculin	Santa Cruz	sc-73614
p21	Cell Signaling Technology	#2947
MCM2	Cell Signaling Technology	#3619
cyclin D1	Abcam	ab18521
p16	Cell Signaling Technology	#80772
phospho-histone H2AX (rabbit)	Cell Signaling Technology	#2566
phospho-histone H2AX (mouse)	Millipore	05-636
histone H3	Abcam	ab18521
H4K20me2	Abcam	ab9052
p53	Santa Cruz	sc-126
FK2	Sigma	04-263
MDC1	Abcam	ab11171
BRCA1	Santa Cruz	sc-6954
53BP1 (rabbit)	Abcam	ab21083
53BP1 (mouse)	Sigma	MAB3802
RIF1	Bethyl Laboratories	A300-569A
p73	Cell Signaling Technology	#14620
BrdU (recognizes CldU)	Abcam	Ab6326
BrdU (recognizes IdU)	BD Biosciences	347580
Anti-mouse IgG HRP	BioRad	170-6516
Anti-rabbit IgG HRP	BioRad	170-6515
Anti-rabbit AlexaFluor488	Thermo Fisher	A11034
Anti-mouse AlexaFluor568	Thermo Fisher	A11031
Anti-rat AlexaFluor555	Thermo Fisher	A21434
Anti-mouse AlexaFluor488	Thermo Fisher	A11029
Anti-rabbit AlexaFluor647	Thermo Fisher	A21245

**Chemicals, peptides, and recombinant proteins**		

Palbociclib	Sigma	PZ0383
Torin1	Sigma	475991
Doxorubicin	Sigma	D1515
Camptothecin	Sigma	208925
Aphidicolin	Sigma	A0781
hydrogen peroxide	Sigma	H1009
KU55933 (ATM inhibitor)	Sigma	1109
AZ20 (ATR inhibitor)	Selleckchem	S7050
nutlin-3a	Sigma	SML0580
CldU	Sigma	C6891
IdU	Sigma	17125
Hoechst 33342	Thermo Fisher	H3570
FxCycle FarRed DNA Stain	Thermo Fisher	F10348
Lipofectamine RNAiMax	Thermo Fisher	13778100
NuPAGE LDS sample buffer (4x)	Thermo Fisher	NP0007
Pierce protease and phosphatase inhibitor mini tablets	Thermo Fisher	A32959
Pierce RIPA lysis buffer	Thermo Fisher	89900
20x MES SDS running buffer	Thermo Fisher	B000102
20x MOPS SDS running buffer	Thermo Fisher	B0002

**Critical commercial assays**		

Click-iT EdU AlexaFluor-488 Flow Cytometry Assay Kit	Thermo Fisher	C10420
Click-iT EdU AlexaFluor-594 Imaging Assay Kit	Thermo Fisher	C10339
Subcellular Protein Fractionation Kit for Cultured Cells	Thermo Fisher	78840
Histone extraction kit	Abcam	ab113476
TMTpro 16plex	Thermo Fisher	44522
CometAssay Electrophoresis Starter Kit	R&D Systems	4250-050-ESK
CellEvent Senescence Green Detection Kit	Thermo Fisher	C10850

**Deposited data**		

TMT proteomics in enlarged/size-constrained cells	This paper	PRIDE: PXD034934

**Experimental models: Cell lines**		

hTERT RPE1 WT	ATCC	CRL-4000
hTERT RPE1 FUCCI	Laboratory of Randy King	N/A
MCF7	ATCC	HTB-22
NALM6	Laboratory of Mike Tyers	N/A

**Oligonucleotides**		

ON-TARGETplus Human TP53	Dharmacon	J-003329-14-0002
ON-TARGETplus Human CDKN1A	Dharmacon	J-003471-09-0002
ON-TARGETplus Human RNF168 SMARTpool	Dharmacon	L-007152-00-0005
On-TARGETplus Human TP73 SMARTpool	Dharmacon	L-0003331-00-0005
siGENOME non-targeting siRNA #5	Dharmacon	D-001210-05-05

**Software and algorithms**		

FIJI	https://imagej.net/software/fiji/	N/A
R	https://www.r-project.org/	N/A
FlowJo	BD Biosciences	N/A
MaxQuant	https://www.maxquant.org	N/A
ShinyGO 0.76	http://bioinformatics.sdstate.edu/go/	N/A

### Resource availability

#### Lead contact

Further information and requests for resources and reagents should be directed to and will be fulfilled by the lead contact, Gabriel Neurohr (gabriel.neurohr@bc.biol.ethz.ch).

#### Materials availability

All cell lines used in this study are available from the lead contact upon request. No unique reagents were generated by this study.

#### Data and code availability

- Proteomic data have been deposited on PRIDE and are publicly available as of the date of publication. Accession numbers are listed in the key resource table. Microscopy and western blotting data reported in this paper are available from the lead contact upon request.
- This paper does not report any original code.
- Any additional information required to reanalyze the data reported in this paper is available from the lead contact upon request.

### Experimental model and study participant details

hTERT-RPE1 and MCF7 cells were purchased from ATCC. hTERT-RPE1 FUCCI cells were a gift from Randall W. King with permission of the RIKEN Institute. NALM6 cells were a gift from Mike Tyers.

### Method details

#### Cell culture, growth conditions, and drug treatments

Cell lines used in this work (hTERT-RPE1 WT, hTERT-RPE1 FUCCI, MCF7, NALM6) were cultured in a humidified incubator at 37°C in the presence of 5% CO_2_.

RPE1 cells were cultured in DMEM/F12 with GlutaMAX (Gibco) + 10% FBS and 1% penicillin/streptomycin. RPE1 cells that were allowed to grow large were seeded at low density (5,000 – 10,000 cells/cm^2^) and treated with 1 μM palbociclib for 6 days. Cells that were allowed to grow large were maintained at subconfluency for the duration of the experiment. RPE1 cells that were size-constrained using Torin1 treatment were seeded at 30,000 cells/cm^2^ prior to treating cells with 1 μM palbociclib + 500 nM Torin1. For contact inhibition experiments, RPE1 cells were seeded at 79,000 cells/cm^2^ for 48 hours before treating cells with 1 μM palbociclib. Note that this is ∼95% confluency after 24 hours. Cells were seeded at this density and allowed to grow for 48 hours because seeding at higher densities caused them to slough off the dish. After 6 days, cells were re-seeded at 30,000 cells/cm^2^ in the presence of 1 μM palbociclib for an additional 24 hours to recover and re-attach. For release experiments, cells were then washed 3x in media and released into media without drugs.

MCF7 cells were cultured in DMEM with GlutaMAX (Gibco) + 10% FBS and 1% penicillin/streptomycin. MCF7 cells were seeded at 30,000 cells/cm^2^ prior to treating cells with 2 μM palbociclib for 6 days. To constrain cell size, MCF7 cells were co-treated with 12 nM Torin1. Note that MCF7 cells are highly sensitive to mTOR inhibition, and higher doses caused significant cell death. For release experiments, cells were washed 3x in media and released into media without drugs.

NALM6 cells were cultured in RPMI-1640 with GlutaMAX (Gibco) + 10% FBS and 1% penicillin/streptomycin. NALM6 cells were maintained between 100,000 cells/mL and 1 x 10^6^ cells/mL. Note that NALM6 cells are sensitive to different FBS sources and doubling time (∼24 hours) should be confirmed for a given FBS source before use.

#### siRNA transfections

Cells were reverse-transfected using Lipofectamine RNAiMax (Invitrogen 13778100) according to manufacturer’s instructions with the following siRNAs at a final concentration of 20 nM. Cells were transfected for 24 hours for all experiments. Knockdowns were confirmed by western blotting. For G_1_ arrest experiments, transfections were carried out in the constant presence of palbociclib.

#### Cell size measurements

Cell size was measured on a Multisizer 4e (Beckman) using Isotone II (Beckman) as a diluent and a 100 μm aperture. For experiments in RPE1 cells, trypsinized cells were resuspended in DMEM/F12 and diluted in 10 mL Isotone II before measuring. MCF7 cells were trypsinized and diluted in Isotone II without re-suspending in media to minimize clumping. NALM6 cells were directly measured in RPMI-1640 diluted in Isotone II. Cell volume distributions were analyzed using a custom R script.

#### Crystal violet staining-based colony formation assays

For colony formation experiments, cells were seeded at ∼260 cells/cm^2^ in the presence of palbociclib (1 μM for RPE1 cells; 2 μM for MCF7 cells) for 24 hours to re-attach. Cells were then gently washed 3x in media and released into fresh media. For DNA damage sensitization experiments in RPE1 cells, cells were released into the indicated concentrations of drugs. Cells were allowed to grow for 10-12 days before washing 1x with ice-cold PBS followed by incubation with ice-cold 100% methanol for 10 minutes on ice. The methanol was aspirated, and then cells were incubated with 0.5% crystal violet (w/v) in 25% methanol at room temperature for 10 minutes. Cells were washed with DI water until clear. Plates were dried at room temperature prior to imaging. Colony formation was quantified using the automated ColonyArea macro in ImageJ.^76^

#### SDS-PAGE and western blotting

Cells were lysed in RIPA lysis buffer (Invitrogen 89900) supplemented with 1x protease/phosphatase inhibitor tablets (Pierce A32959) by periodic vortexing on ice. Lysates were clarified by centrifugation at 16,000 x *g* for 10 minutes at 4°C. Lysates were transferred to fresh tubes, and protein concentrations were measured using a BCA assay kit according to manufacturer’s instructions (Pierce 23225). Lysates were combined with 4x LDS sample buffer (Invitrogen NP0007) + 25 mM DTT to a final concentration of 1x and were boiled at 95°C for 10 minutes. Equal masses of protein (except for histone-normalized experiments, where lysate was derived from an equal number of cells) were loaded on Bolt 4-12% Bis-Tris gels (Invitrogen) and resolved in either 1x MES or MOPS SDS running buffer (Invitrogen B000102, B0002). Proteins were transferred to PVDF membranes at 250 mA at 4°C for 1 hr 15 min using a wet transfer apparatus. Membranes were blocked in 5% milk in TBS-T before incubating with primary antibodies in 5% milk in TBS-T at 4°C with agitation overnight. Membranes were washed 3x in TBS-T for ∼10 minutes each, incubated with secondary mouse or rabbit IgG HRP-conjugated antibodies in 5% milk in TBS-T for 45-60 minutes, washed again, and then developed using enhanced chemiluminescent substrate solutions as described by the manufacturer (Thermo 34095, Thermo 34578).

#### Chromatin fractionation

Isolation of chromatin bound MCM2 and PCNA was carried out using a subcellular fractionationation kit for cultured cells (Thermo 78840) according to the manufacturer’s protocol. Isolated fractions were diluted to 1x in LDS sample buffer (Invitrogen NP0007) + 25 mM DTT and were analyzed by SDS-PAGE and western blotting as described above.

#### Histone extraction

Isolation of histones was carried out using a histone acid extraction kit for cultured cells (Abcam AB113476). Isolated histone fractions were diluted to 1x in LDS sample buffer (Invitrogen NP0007) + 25 mM DTT and were analyzed by SDS-PAGE and western blotting as described.

#### Alkaline comet assay (single cell electrophoresis)

Cells were collected with trypsin and washed once in PBS. Cells were then suspended in PBS at approximately 1 x 10^6^ cells/mL and were combined in a 1:30 ratio with CometAssay LMAgarose (R&D Systems 4250-050-02) at 37°C. 60 μL of cell suspension were spread in an even layer on CometSlides (R&D Systems 4250-200-03) and allowed to polymerize at 4°C in a humidified chamber for 30 minutes. Slides were then immersed in pre-chilled lysis buffer (R&D Systems 4250-050-01) overnight. DNA unwinding was performed at room temperature for 1 hour in 300 mM NaOH/3 mM EDTA solution. Following unwinding, electrophoresis was performed at 4°C in prechilled 300 mM NaOH/3 mM EDTA using the CometAssay Electrophoresis System II (R&D Systems, 4250-050-ES) for 30 minutes at 18V/330mA. Slides were then washed 2x in PBS for 5 minutes each. Slides were fixed in -20°C 70% ethanol for 30 minutes. Slides were then washed 2x in PBS for 10 minutes each and 2x briefly in MilliQ water, after which they were then dried at 37°C. DNA was stained using 1:5000 SYBR Gold (Invitrogen S11494) for 30 minutes in the dark. Slides were then washed 2x in PBS for 10 minutes and 2x briefly with MilliQ water before drying again. Coverslips were affixed to slides using clear nail polish before imaging with a Nikon Ti2-E inverted microscope using a 20x/NA 0.75 air objective. Images were processed and tail moments— calculated as (tail length) x (% DNA in the tail— were calculated using the automated OpenComet ImageJ plugin.^77^ Mis-segmented comets were manually removed from the analysis.

#### DNA Fiber Analysis

The DNA fiber analysis protocol was adapted from previous work.^78^ Enlarged and size-constrained cells were released from palbociclib for 14 hours. Cells were pulse labeled with 25 μM CldU for 15 minutes at 37°C and then with 250 μM CO_2_ equilibrated IdU for 15 minutes at 37°C. After labeling, cells were washed in ice-cold PBS, trypsinized, and re-suspended in PBS at between 1-1.5 x 10^6^ cells/mL. 2.5 μL of cell suspension was placed on a glass slide and allowed to dry slightly at RT for 5 minutes, after which spreading buffer (200 mM Tris pH 7.4, 50 mM EDTA, 0.5% SDS) was mixed with the sample and incubated for 2 minutes at room temperature. Slides were then tilted to allow DNA to spread and left to air-dry for 2 minutes. Slides were then incubated in 3:1 methanol:acetic acid in a coplin jar for 10 minutes, after which they were air-dried and stored at 4°C until immunostaining.

For immunostaining, slides were rinsed twice with water and denatured in 2.5M HCl for 1 hour and 15 minutes. Slides were then rinsed twice in PBS and blocked for 1 hour in blocking solution (1% BSA in PBS + 0.1% Tween-20). Slides were then stained overnight with primary antibodies (rat anti-BrdU which recognizes CldU, 1:250 and mouse anti-BrdU which recognizes IdU, 1:100). Slides were rinsed 3x in PBS followed by a 10-minute incubation in 4% paraformaldehyde. Slides were then rinsed 3x in PBS and stained with secondary antibodies (1:500 anti-rat AlexaFluor555 and 1:500 anti-mouse AlexaFluor488) for 1.5 hours at room temperature in the dark. Slides were washed twice in PBS, and coverslips were mounted using Fluoroshield mounting medium

Images were collected with a Leica TCS SPE2 confocal microscope controlled by LAS (Leica) using a 63x/NA 1.30 oil objective lens. AlexaFluor488 and AlexaFluor555 fluorescence was excited with a Leica EL6000 Metal Halide light source containing an Osram HXP bulb. Images were processed using Fiji. 85-200 fibers were counted for each experiment. Maximum projections of the composite images were constructed to visualize red and green channels. The ‘line’ tool in Fiji was used to measure the length of ongoing fibers, represented by consecutive red and green tracks. Percentage of origins firing was calculated as (1^st^ + 2^nd^ pulse origins) / (ongoing forks + origins + terminations) x 100. Origin firing rates were calculated as a weighted average from three independent experimental replicates.

#### Time lapse fluorescence imaging

G_1_ arrested cells were plated in the presence of palbociclib on an 8-well coverslip dish (Ibidi 80826) to be approximately 50-70% confluent after 24 hours. Note that cell cycle re-entry dynamics are highly dependent on cell density at the time of release since RPE1 cells are subject to contact inhibition. For siRNA transfection experiments, cells were transfected at the time of plating on coverslip dishes. For cell cycle release experiments, compounds (and siRNAs) were washed out and cells were imaged immediately following the addition of fresh media. Coverslips were inserted into a covered cage microscope incubator (Okolabs) with temperature and humidity control at 37°C and 5% CO_2_ and mounted on a motorized microscope stage (Prior ProScan HLD117NN). All images were collected on a Nikon Ti motorized inverted microscope equipped with a piezo z-drive (Prior), a 20x/0.75 NA S Fluor air objective lens, and the Perfect Focus system. mCherry fluorescence was excited with a Lumencor Spectra III light engine using a 578/21 excitation filter and a 641/75 emission filter (Semrock). mAG1 fluorescence was excited using a 474/27 excitation filter and a 515/30 emission filter (Semrock). Images were acquired with a Hamamatsu ORCA Fusion BT camera controlled with Nikon Elements image acquisition software. Four fields of view were collected per condition, and brightfield and/or fluorescence images were captured at 5- to 6-minute intervals.

#### Cell fixation and staining

##### Immunostaining and imaging

Cells were seeded onto glass coverslips and treated as indicated. Cells were then fixed with 4% formaldehyde solution for 10 min and permeabilized with 0.25% Triton X-100 in PBS for 10 min. Fixed cells were washed three times for 5 minutes with 0.05% Triton X-100 in PBS and blocked with 1% BSA in PBS for 30 min at room temperature. Coverslips were incubated with primary antibodies for one hour at room temperature and were then washed three times for 5 minutes with 0.05% Triton X-100 in PBS. For detection of the primary antibodies, cells were incubated with the appropriate AlexaFluor-conjugated secondary antibodies in blocking solution for 1 hour at room temperature. Cells were then washed once with 0.05% Triton X-100 in PBS for 5 minutes and washed twice with 0.05% Triton X-100 + Hoechst 33342 (0.1 μg/mL) in PBS for 5 minutes. Coverslips were washed briefly with Milli-Q water and mounted onto glass slides using either Vectashield mounting medium (Vector Laboratories) or ProlongDiamond Antifade mounting medium (Thermo P36965).

Fixed cell microscopy was performed on a Nikon Eclipse Ti-E inverted microscope using a 60x/NA 1.40 oil objective. Hoechst fluorescence was excited with a Lumencor SpectraX light engine using a 390/18 nm or 378/52 nm excitation filter and a 460/50 nm or 432/36 nm emission filter. AlexaFluor488 fluorescence was excited using a 470/40 nm or 474/27 nm excitation filter and a 520/35 nm or 515/30 nm emission filter. AlexaFluor568 fluorescence was excited using a 575/27 nm or 578/21 nm excitation filter and a 641/75 nm or 641/75 nm emission filter. AlexaFluor647 fluorescence was excited using a 635/18 nm excitation filter and a 698/70 emission filter. Images were acquired with a Hamamatsu ORCA Flash 4.0 camera or a Hamamatsu ORCA Fusion BT camera controlled by ImageJ μManager^79^ or NIS (Nikon) Elements. Five to ten fields of view were collected per condition.

Nuclear foci formation for DNA damage proteins (e.g., γH2AX, 53BP1, MDC1, RIF1, BRCA1) was analyzed manually using the ROI manager tool in ImageJ. Nuclei were segmented based on Hoechst staining, and individual cells were quantified in a single, focused Z-plane for each cell.

For imaging experiments measuring mean intensity, nuclei were segmented based on Hoechst staining, and mean intensities were measured and background-subtracted for individual nuclei in a single, focused Z-plane for each cell.

##### EdU staining and flow cytometry-based detection

Cells were incubated with 10 μM EdU-alkyne under the indicated conditions. For flow cytometry, cells were then collected, fixed, and labeled with AlexaFluor-488-azide according to the manufacturer’s protocol (Thermo C10632). EdU incorporation was analyzed using a BD FACSCanto cell analyzer. Cells were co-stained for DNA using FxCycle FarRed (Thermo F10348) in the presence of RNase A to gate for single cells. Note that this experimental setup was not viable in RPE1 cells due to cell lysis during fixation and sample preparation for flow cytometry. EdU incorporation was measured in RPE1 cells by fluorescence microscopy. Cells were grown on coverslips as indicated before fixing in 4% paraformaldehyde and labeling with AlexaFluor-594-azide according to the manufacturer’s protocol (Thermo C10339). Cells were counterstained with Hoechst 33342, and images were acquired with a Nikon Ti2-E inverted microscope using a 20x/NA 0.75 air objective.

##### Fixed cell imaging of nuclear abnormalities

For analysis of nuclear defects in MCF7 cells, enlarged and size-constrained MCF7 cells were seeded in a black-walled 96-well plate (Corning 3606) in the presence of palbociclib for 24 hours. Cells were then washed 3x with media and released from drug treatment for two days. Cells were then fixed (10% formalin and 0.1% Triton X-100 in PBS) and DNA was stained with Hoechst 33342 (1 μg/mL) for 45 minutes in the dark before imaging with an ImageXpress Micro high content microscope (Molecular Devices) equipped with a 20x objective and the DAPI filter. At least 15 images were collected and analyzed for each condition.

#### TMT mass spectrometry sample preparation

Samples were prepared essentially as described previously^80^: Cells were cultured as described in biological triplicate. Cell pellets were re-suspended in urea lysis buffer containing 8 M urea, 200 mM EPPS pH 8.0, and EDTA-free Pierce protease and phosphatase inhibitor tablets (Thermo Fisher Scientific, A32961). Lysates were passed through a 21-gauge needle 20 times, and protein concentrations were measured by BCA assay (Thermo Fisher Scientific). One hundred micrograms of protein were reduced with 5 mM tris-2-carboxyethyl-phosphine (TCEP) at room temperature for 15 min, alkylated with 10 mM iodoacetamide at room temperature for 30 min in the dark, and were quenched with 15 mM DTT for 15 min at room temperature. Proteins were precipitated using a methanol/chloroform extraction. Pelleted proteins were resuspended in 100 μL 200 mM EPPS, pH 8.0. LysC (Wako 125-05061) was added at a 1:50 enzyme/protein ratio, and samples were incubated overnight at room temperature with agitation. Following overnight incubation, trypsin (Promega V5111) was added at a 1:100 enzyme/protein ratio, and samples were incubated for an additional 6 h at 37 °C. Tryptic digestion was halted by the addition of acetonitrile (ACN). Tandem mass tag (TMT) isobaric reagents (TMTpro 16plex Thermo Fisher Scientific 44522) were dissolved in anhydrous ACN to a final concentration of 20 mg/mL, of which a unique TMT label was added at a 2:1 label:peptide ratio. Peptides were incubated at room temperature for one hour with vortexing at the 30-minute interval. TMT labeling reactions were quenched by the addition of 10 μL of 5% hydroxylamine. Equal amounts of each sample were combined at a 1:1 ratio across all channels and dried by vacuum centrifugation. Samples were re-suspended in 1% formic acid and desalted using a 50 mg 1 cc SepPak C18 cartridge (Waters WAT054955) following manufacture’s instruction. Briefly, peptides were washed with 5% ACN and 0.1% formic acid, eluted with 50% ACN and 0.1% formic acid and dried. Subsequently, peptides were subjected to fractionation with basic pH reverse phase HPLC chromatography using a linear gradient (5-40% ACN, 9 mM ammonium bicarbonate) on XBridge peptide BEH C18 column (130 Å, 3.5 μm, 4.6 mm X 250 mm, Waters). Fractions were collected in 96-well format plate and consolidated on 12 fractions, dried, and re-suspended in 5% ACN and 5% formic acid for LC-MS/MS processing.

#### TMT Mass Spectrometry Analysis

LC-MS/MS analysis was performed on an Orbitrap Fusion Lumos Tribrid mass spectrometer (Thermo Scientific) coupled to an Acquity UPLC M-class system (Waters). Peptides were loaded on a commercial trap column (Symmetry C18, 100Å, 5 μm, 180 μm^∗^20mm, Waters) and separated on a commercial column (HSS T3, 100Å, 1.8 μm, 75 μm^∗^250mm, Waters) using a 113 min gradient from 5% to 35% acetonitrile at a flow rate of 300 nL/min. The mass spectrometer was operated in data dependent acquisition (DDA) mode with 2s cycle time. MS1 data were collected in the Orbitrap (400-1400 m/z) at 60’000 resolution, 50 ms injection time and 4e5 AGC target. Ions with charge states between two and six were isolated in quadrupole (isolation window 0.5 m/z), fragmented (CID, NCE 35%) and MS2 scans were collected in the ion trap (Turbo, maximum injection time 120 ms, AGC 1.5e4); 60 s of dynamic exclusion was used. MS3 quantification scans were performed with ten notches; ions were isolated in the quadrupole (2 m/z), fragmented (HCD, NCE 45%) and identified in the Orbitrap (50’000 resolution, maximum injection time 86 ms and AGC 2e5).

### Quantification and statistical analysis

#### Mass Spectrometry Data Analysis

Acquired spectra were searched using the MaxQuant software package version 2.1.0.0 embedded with the Andromeda search engine^81^ against the human proteome reference dataset (http://www.uniprot.org/, downloaded on 06.04.2021) extended with reverse decoy sequences. The search parameters were set to include only full tryptic peptides (Trypsin/P), a maximum of two missed cleavage sites, carbamidomethyl and TMT16 as static peptide modification, oxidation (M) and acetylation (Protein N-term). Precursor and fragment ion tolerance was set respectively to 4.5ppm and 20ppm. A false discovery rate (FDR) of <1% was used at the PSM and protein level. Reporter intensities for proteins identified with at least 2 peptides (5884) were normalized, and missing values (1.7%) were imputed using random sampling from a normal distribution generated from 1% less intense values. ANOVA statistical tests were performed to compare protein profiles in all conditions. *P*-values were corrected using the Benjamini-Hochberg method.^82^ Matrices with protein intensities are reported in Table S1.

#### Gene Enrichment Analysis

Gene enrichment analysis of the subset of proteins identified in the proteomics with a significant (adj. p-value < 0.05) increase in abundance with a log_2_(FC) ≥ 1 using both size-constraint methods was performed using the ShinyGO tool for biological processes^83^ using the total proteome as a background.

#### Statistical Methods

Statistical testing was carried out using the strategies outlined in each figure legend. For all figures, the following p-value indication scheme is used: ns: p > 0.05; ^∗^: p < 0.05; ^∗∗^: p < 0.01; ^∗∗∗^: p < 0.001; ^∗∗∗∗^: p < 0.0001.

## Supplemental information

Document S1. Figures S1–S6

Table S1. TMT proteomics (full table), related to Figures 2 and 3

Table S2. Decreasing proteins in TMT proteomics, related to Figure 3

Table S3. Increasing proteins in TMT proteomics, related to Figure 3

Document S2. Article plus supplemental information

## References

[1] Campisi J., d'Adda di Fagagna F. (2007). Cellular senescence: when bad things happen to good cells. Nat. Rev. Mol. Cell Biol..

[2] Collado M., Blasco M.A., Serrano M. (2007). Cellular senescence in cancer and aging. Cell.

[3] Wang L., Lankhorst L., Bernards R. (2022). Exploiting senescence for the treatment of cancer. Nat. Rev. Cancer.

[4] Yang L.X., Fang J., Chen J.D. (2017). Tumor cell senescence response produces aggressive variants. Cell Death Discov..

[5] Hayflick L., Moorhead P.S. (1961). The serial cultivation of human diploid cell strains. Exp. Cell Res..

[6] Demidenko Z.N., Blagosklonny M.V. (2008). Growth stimulation leads to cellular senescence when the cell cycle is blocked. Cell Cycle.

[7] Neurohr G.E., Terry R.L., Lengefeld J., Bonney M., Brittingham G.P., Moretto F., Miettinen T.P., Vaites L.P., Soares L.M., Paulo J.A. (2019). Excessive cell growth causes cytoplasm dilution and contributes to senescence. Cell.

[8] Lengefeld J., Cheng C.W., Maretich P., Blair M., Hagen H., McReynolds M.R., Sullivan E., Majors K., Roberts C., Kang J.H. (2021). Cell size is a determinant of stem cell potential during aging. Sci. Adv..

[9] Wilson G.A., Vuina K., Sava G., Huard C., Meneguello L., Coulombe-Huntington J., Bertomeu T., Maizels R.J., Lauring J., Kriston-Vizi J. (2023). Active growth signaling promotes senescence and cancer cell sensitivity to CDK7 inhibition. Mol. Cell.

[10] Crozier L., Foy R., Mouery B.L., Whitaker R.H., Corno A., Spanos C., Ly T., Gowen Cook J., Saurin A.T. (2022). CDK4/6 inhibitors induce replication stress to cause long-term cell cycle withdrawal. EMBO J..

[11] Lanz M.C., Zatulovskiy E., Swaffer M.P., Zhang L., Zhang S., You D.S., Marinov G., McAlpine P., Elias J.E., Skotheim J.M. (2022). Increasing cell size remodels the proteome and promotes senescence. Mol. Cell.

[12] Zadrag-Tecza R., Kwolek-Mirek M., Bartosz G., Bilinski T. (2009). Cell volume as a factor limiting the replicative lifespan of the yeast Saccharomyces cerevisiae. Biogerontology.

[13] Engeland K. (2022). Cell cycle regulation: p53-p21-RB signaling. Cell Death Differ..

[14] Zhang H.S., Postigo A.A., Dean D.C. (1999). Active transcriptional repression by the Rb–E2F complex mediates G1 arrest triggered by p16INK4a, TGFβ, and contact inhibition. Cell.

[15] Kastenhuber E.R., Lowe S.W. (2017). Putting p53 in context. Cell.

[16] Hinchcliffe E.H., Day C.A., Karanjeet K.B., Fadness S., Langfald A., Vaughan K.T., Dong Z. (2016). Chromosome missegregation during anaphase triggers p53 cell cycle arrest through histone H3.3 Ser31 phosphorylation. Nat. Cell Biol..

[17] López-García C., Sansregret L., Domingo E., McGranahan N., Hobor S., Birkbak N.J., Horswell S., Grönroos E., Favero F., Rowan A.J. (2017). BCL9L dysfunction impairs caspase-2 expression permitting aneuploidy tolerance in colorectal cancer. Cancer Cell.

[18] Thompson S.L., Bakhoum S.F., Compton D.A. (2010). Mechanisms of chromosomal instability. Curr. Biol..

[19] Liu D., Xu Y. (2011). p53, oxidative stress, and aging. Antioxid. Redox Signal..

[20] Abbas T., Dutta A. (2009). p21 in cancer: intricate networks and multiple activities. Nat. Rev. Cancer.

[21] Hsieh J.-K., Chan F.S.G., O’Connor D.J., Mittnacht S., Zhong S., Lu X. (1999). RB regulates the stability and the apoptotic function of p53 via MDM2. Mol. Cell.

[22] Sakaue-Sawano A., Kurokawa H., Morimura T., Hanyu A., Hama H., Osawa H., Kashiwagi S., Fukami K., Miyata T., Miyoshi H. (2008). Visualizing spatiotemporal dynamics of multicellular cell-cycle progression. Cell.

[23] Leontieva O.V., Blagosklonny M.V. (2010). DNA damaging agents and p53 do not cause senescence in quiescent cells, while consecutive re-activation of mTOR is associated with conversion to senescence. Aging (Albany, NY).

[24] Korotchkina L.G., Leontieva O.V., Bukreeva E.I., Demidenko Z.N., Gudkov A.V., Blagosklonny M.V. (2010). The choice between p53-induced senescence and quiescence is determined in part by the mTOR pathway. Aging (Albany, NY).

[25] Futreal P.A., Barrett J.C. (1991). Failure of senescent cells to phosphorylate the RB protein. Oncogene.

[26] Neumann F.R., Nurse P. (2007). Nuclear size control in fission yeast. J. Cell Biol..

[27] Huber M.D., Gerace L. (2007). The size-wise nucleus: nuclear volume control in eukaryotes. J. Cell Biol..

[28] Spies J., Lukas C., Somyajit K., Rask M.B., Lukas J., Neelsen K.J. (2019). 53BP1 nuclear bodies enforce replication timing at under-replicated DNA to limit heritable DNA damage. Nat. Cell Biol..

[29] Blackford A.N., Jackson S.P. (2017). ATM, ATR, and DNA-PK: the trinity at the heart of the DNA damage response. Mol. Cell.

[30] Georgakilas A.G., Martin O.A., Bonner W.M. (2017). p21: A two-faced genome guardian. Trends Mol. Med..

[31] Crozier L., Foy R., Adib R., Kar A., Holt J.A., Pareri A.U., Valverde J.M., Rivera R., Weston W.A., Wilson R. (2023). CDK4/6 inhibitor-mediated cell overgrowth triggers osmotic and replication stress to promote senescence. Mol. Cell.

[32] Wang B., Varela-Eirin M., Brandenburg S.M., Hernandez-Segura A., van Vliet T., Jongbloed E.M., Wilting S.M., Ohtani N., Jager A., Demaria M. (2022). Pharmacological CDK4/6 inhibition reveals a p53-dependent senescent state with restricted toxicity. EMBO J..

[33] Canman C.E. (2001). Replication checkpoint: preventing mitotic catastrophe. Curr. Biol..

[34] Johmura Y., Shimada M., Misaki T., Naiki-Ito A., Miyoshi H., Motoyama N., Ohtani N., Hara E., Nakamura M., Morita A. (2014). Necessary and sufficient role for a mitosis skip in senescence induction. Mol. Cell.

[35] Krenning L., Feringa F.M., Shaltiel I.A., van den Berg J., Medema R.H. (2014). Transient activation of p53 in G2 phase is sufficient to induce senescence. Mol. Cell.

[36] Moeglin E., Desplancq D., Conic S., Oulad-Abdelghani M., Stoessel A., Chiper M., Vigneron M., Didier P., Tora L., Weiss E. (2019). Uniform widespread nuclear phosphorylation of histone H2AX is an indicator of lethal DNA replication stress. Cancers.

[37] Ge X.Q., Jackson D.A., Blow J.J. (2007). Dormant origins licensed by excess Mcm2-7 are required for human cells to survive replicative stress. Genes Dev..

[38] Woodward A.M., Göhler T., Luciani M.G., Oehlmann M., Ge X., Gartner A., Jackson D.A., Blow J.J. (2006). Excess Mcm2-7 license dormant origins of replication that can be used under conditions of replicative stress. J. Cell Biol..

[39] Zirkel A., Nikolic M., Sofiadis K., Mallm J.P., Brackley C.A., Gothe H., Drechsel O., Becker C., Altmüller J., Josipovic N. (2018). HMGB2 loss upon senescence entry disrupts genomic organization and induces CTCF clustering across cell types. Mol. Cell.

[40] Aird K.M., Iwasaki O., Kossenkov A.V., Tanizawa H., Fatkhutdinov N., Bitler B.G., Le L., Alicea G., Yang T.L., Johnson F.B. (2016). HMGB2 orchestrates the chromatin landscape of senescence-associated secretory phenotype gene loci. J. Cell Biol..

[41] Freund A., Laberge R.M., Demaria M., Campisi J. (2012). Lamin B1 loss is a senescence-associated biomarker. Mol. Biol. Cell.

[42] Shimi T., Butin-Israeli V., Adam S.A., Hamanaka R.B., Goldman A.E., Lucas C.A., Shumaker D.K., Kosak S.T., Chandel N.S., Goldman R.D. (2011). The role of nuclear lamin B1 in cell proliferation and senescence. Genes Dev..

[43] Claude K.L., Bureik D., Chatzitheodoridou D., Adarska P., Singh A., Schmoller K.M. (2021). Transcription coordinates histone amounts and genome content. Nat. Commun..

[44] McKinley K.L., Cheeseman I.M. (2017). Large-scale analysis of CRISPR/Cas9 cell-cycle knockouts reveals the diversity of p53-dependent responses to cell-cycle defects. Dev. Cell.

[45] Marusyk A., Wheeler L.J., Mathews C.K., DeGregori J. (2007). p53 mediates senescence-like arrest induced by chronic replicational stress. Mol. Cell. Biol..

[46] Taylor W.R., Agarwal M.L., Agarwal A., Stacey D.W., Stark G.R. (1999). p53 inhibits entry into mitosis when DNA synthesis is blocked. Oncogene.

[47] Shiloh Y. (2006). The ATM-mediated DNA-damage response: taking shape. Trends Biochem. Sci..

[48] Dungrawala H., Rose K.L., Bhat K.P., Mohni K.N., Glick G.G., Couch F.B., Cortez D. (2015). The replication checkpoint prevents two types of fork collapse without regulating replisome stability. Mol. Cell.

[49] Ribeyre C., Zellweger R., Chauvin M., Bec N., Larroque C., Lopes M., Constantinou A. (2016). Nascent DNA proteomics reveals a chromatin remodeler required for topoisomerase I loading at replication forks. Cell Rep..

[50] Mukherjee C., Tripathi V., Manolika E.M., Heijink A.M., Ricci G., Merzouk S., de Boer H.R., Demmers J., van Vugt M.A.T.M., Ray Chaudhuri A. (2019). RIF1 promotes replication fork protection and efficient restart to maintain genome stability. Nat. Commun..

[51] Audoynaud C., Vagner S., Lambert S. (2021). Non-homologous end-joining at challenged replication forks: an RNA connection?. Trends Genet..

[52] Wang B., Matsuoka S., Carpenter P.B., Elledge S.J. (2002). BP1, a mediator of the DNA damage checkpoint. Science.

[53] Panier S., Boulton S.J. (2014). Double-strand break repair: 53BP1 comes into focus. Nat. Rev. Mol. Cell Biol..

[54] Kilic S., Lezaja A., Gatti M., Bianco E., Michelena J., Imhof R., Altmeyer M. (2019). Phase separation of 53BP1 determines liquid-like behavior of DNA repair compartments. EMBO J..

[55] Cuella-Martin R., Oliveira C., Lockstone H.E., Snellenberg S., Grolmusova N., Chapman J.R. (2016). 53BP1 integrates DNA repair and p53-dependent cell fate decisions via distinct mechanisms. Mol. Cell.

[56] Ghodke I., Remisova M., Furst A., Kilic S., Reina-San-Martin B., Poetsch A.R., Altmeyer M., Soutoglou E. (2021). AHNAK controls 53BP1-mediated p53 response by restraining 53BP1 oligomerization and phase separation. Mol. Cell.

[57] Stewart G.S., Wang B., Bignell C.R., Taylor A.M., Elledge S.J. (2003). MDC1 is a mediator of the mammalian DNA damage checkpoint. Nature.

[58] Doil C., Mailand N., Bekker-Jensen S., Menard P., Larsen D.H., Pepperkok R., Ellenberg J., Panier S., Durocher D., Bartek J. (2009). RNF168 binds and amplifies ubiquitin conjugates on damaged chromosomes to allow accumulation of repair proteins. Cell.

[59] Stewart G.S., Panier S., Townsend K., Al-Hakim A.K., Kolas N.K., Miller E.S., Nakada S., Ylanko J., Olivarius S., Mendez M. (2009). The RIDDLE syndrome protein mediates a ubiquitin-dependent signaling cascade at sites of DNA damage. Cell.

[60] Botuyan M.V., Lee J., Ward I.M., Kim J.-E., Thompson J.R., Chen J., Mer G. (2006). Structural basis for the methylation state-specific recognition of histone H4-K20 by 53BP1 and Crb2 in DNA repair. Cell.

[61] Sanders S.L., Portoso M., Mata J., Bähler J., Allshire R.C., Kouzarides T. (2004). Methylation of histone H4 lysine 20 controls recruitment of Crb2 to sites of DNA damage. Cell.

[62] Chapman J.R., Barral P., Vannier J.B., Borel V., Steger M., Tomas-Loba A., Sartori A.A., Adams I.R., Batista F.D., Boulton S.J. (2013). RIF1 is essential for 53BP1-dependent nonhomologous end joining and suppression of DNA double-strand break resection. Mol. Cell.

[63] Leontieva O.V., Demidenko Z.N., Blagosklonny M.V. (2014). Contact inhibition and high cell density deactivate the mammalian target of rapamycin pathway, thus suppressing the senescence program. Proc. Natl. Acad. Sci. USA.

[64] Zatulovskiy E., Lanz M.C., Zhang S., McCarthy F., Elias J.E., Skotheim J.M. (2022). Delineation of proteome changes driven by cell size and growth rate. Front. Cell Dev. Biol..

[65] Michaloglou C., Crafter C., Siersbaek R., Delpuech O., Curwen J.O., Carnevalli L.S., Staniszewska A.D., Polanska U.M., Cheraghchi-Bashi A., Lawson M. (2018). Combined inhibition of mTOR and CDK4/6 is required for optimal blockade of E2F function and long-term growth inhibition in estrogen receptor-positive breast cancer. Mol. Cancer Ther..

[66] Yang J., Dungrawala H., Hua H., Manukyan A., Abraham L., Lane W., Mead H., Wright J., Schneider B.L. (2011). Cell size and growth rate are major determinants of replicative lifespan. Cell Cycle.

[67] Anglada T., Genescà A., Martín M. (2020). Age-associated deficient recruitment of 53BP1 in G1 cells directs DNA double-strand break repair to BRCA1/CtIP-mediated DNA-end resection. Aging (Albany, NY).

[68] Oh S., Lee C., Yang W., Li A., Mukherjee A., Basan M., Ran C., Yin W., Tabin C.J., Fu D. (2022). Protein and lipid mass concentration measurement in tissues by stimulated Raman scattering microscopy. Proc. Natl. Acad. Sci. USA.

[69] Liu X., Oh S., Kirschner M.W. (2022). The uniformity and stability of cellular mass density in mammalian cell culture. Front. Cell Dev. Biol..

[70] Zhang L., Geng X., Wang F., Tang J., Ichida Y., Sharma A., Jin S., Chen M., Tang M., Pozo F.M. (2022). 53BP1 regulates heterochromatin through liquid phase separation. Nat. Commun..

[71] Mycock K., Zhan L., Taylor-Stokes G., Milligan G., Mitra D. (2021). Real-World palbociclib use in HR+/HER2- advanced breast cancer in Canada: the IRIS Study. Curr. Oncol..

[72] Goel S., DeCristo M.J., McAllister S.S., Zhao J.J. (2018). CDK4/6 inhibition in cancer: beyond cell cycle arrest. Trends Cell Biol..

[73] Foy R., Crozier L., Pareri A.U., Valverde J.M., Park B.H., Ly T., Saurin A.T. (2023). Oncogenic signals prime cancer cells for toxic cell overgrowth during a G1 cell cycle arrest. Mol. Cell.

[74] Maskey R.S., Wang F., Lehman E., Wang Y., Emmanuel N., Zhong W., Jin G., Abraham R.T., Arndt K.T., Myers J.S., Mazurek A. (2021). Sustained mTORC1 activity during palbociclib-induced growth arrest triggers senescence in ER+ breast cancer cells. Cell Cycle.

[75] Biran A., Zada L., Abou Karam P., Vadai E., Roitman L., Ovadya Y., Porat Z., Krizhanovsky V. (2017). Quantitative identification of senescent cells in aging and disease. Aging Cell.

[76] Guzmán C., Bagga M., Kaur A., Westermarck J., Abankwa D. (2014). ColonyArea: an ImageJ plugin to automatically quantify colony formation in clonogenic assays. PLoS One.

[77] Gyori B.M., Venkatachalam G., Thiagarajan P.S., Hsu D., Clement M.-V. (2014). OpenComet: an automated tool for comet assay image analysis. Redox Biol..

[78] Petermann E., Woodcock M., Helleday T. (2010). Chk1 promotes replication fork progression by controlling replication initiation. Proc. Natl. Acad. Sci. USA.

[79] Edelstein A.D., Tsuchida M.A., Amodaj N., Pinkard H., Vale R.D., Stuurman N. (2014). Advanced methods of microscope control using μManager software. J. Biol. Methods.

[80] Manohar S., Yu Q., Gygi S.P., King R.W. (2020). The insulin receptor adaptor IRS2 is an APC/C substrate that promotes cell cycle protein expression and a robust spindle assembly checkpoint. Mol. Cell. Proteomics.

[81] Cox J., Mann M. (2008). MaxQuant enables high peptide identification rates, individualized p.p.b.-range mass accuracies and proteome-wide protein quantification. Nat. Biotechnol..

[82] Benjamini Y., Hochberg Y. (1995). Controlling the false discovery rate – a practical and powerful approach to multiple testing. J. R. Stat. Soc. B.

[83] Ge S.X., Jung D., Yao R. (2020). ShinyGO: a graphical gene-set enrichment tool for animals and plants. Bioinformatics.

