# Supplementary material for: Genome homeostasis defects drive enlarged cells into senescence
Source: Mol Cell. 2023 Nov 16;83(22):4032–4046.e6. doi: 10.1016/j.molcel.2023.10.018 (PMC10659931; doi:10.1016/j.molcel.2023.10.018)
Supplement: Document S1. Figures S1–S6 [file mmc1.pdf]

Molecular Cell, Volume *83*

Supplemental information

Genome homeostasis defects drive enlarged cells into senescence

Sandhya Manohar, Marianna E. Estrada, Federico Uliana, Karla Vuina, Patricia Moyano Alvarez, Robertus A.M. de Bruin, and Gabriel E. Neurohr

Supplemental Materials for

Genome homeostasis defects drive enlarged cells into senescence

Sandhya Manohar [a], Marianna E. Estrada [a], Federico Uliana [a], Karla Vuina [b], Patricia Moyano Alvarez [a], Robertus A.M. de Bruin [b,c], and Gabriel E. Neurohr [a,e*]

[a] Institute for Biochemistry, Department of Biology, ETH Zürich, Zürich, Zürich 8093 Switzerland

[b] Laboratory for Molecular Cell Biology, University College London, London, WC1E 6BT United Kingdom

[c] UCL Cancer Institute, University College London, London, WC1E 6BT United Kingdom

[e] Lead contact

* Correspondence: gabriel.neurohr@bc.biol.ethz.ch

This PDF file includes:

Figure S1 to S6

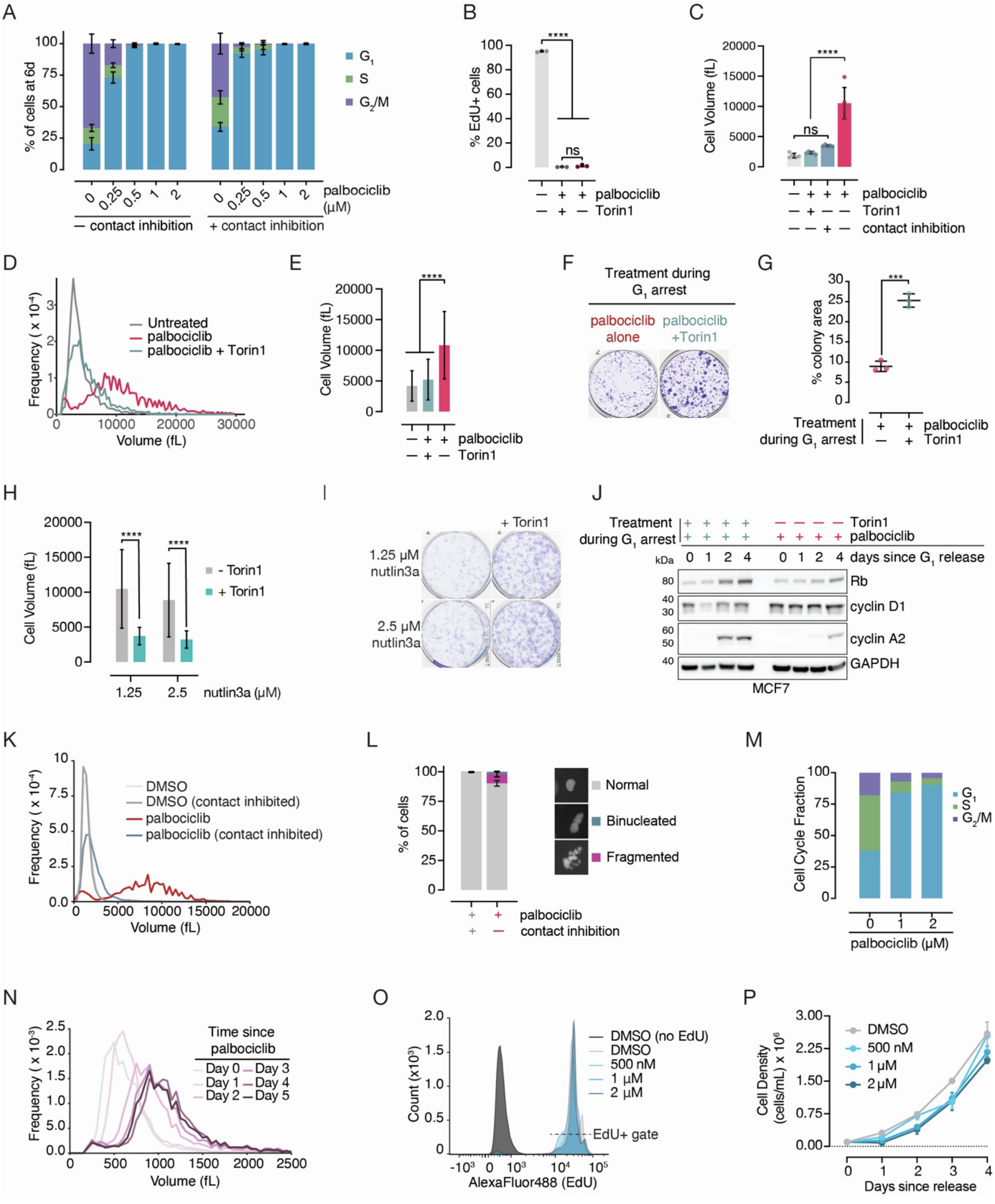

Figure S1: *Additional data related to Figure 1.*

(**A**) Cell cycle distributions (based on FUCCI reporters) for RPE1 FUCCI cells treated with various doses of palbociclib (+/- contact inhibition) for 6 days + 1 day of recovery in the presence of palbociclib. Four images were analyzed for each replicate with a total of at least 240 cells scored per replicate. Error bars = mean ± SD of three replicates. 1 μM palbociclib was used for all of the subsequent RPE1 experiments in this study.

(**B**) RPE1 WT cells were treated with DMSO or 1 µM palbociclib (+/- Torin1) for 6 days + 1 day of recovery in the presence of palbociclib alone. Cells were then treated with 10 µM EdU for an additional 24 hours while maintaining 1 µM palbociclib. Cells were then fixed and EdU was derivatized with AlexaFluor594 azide. EdU incorporation was measured by fluorescence microscopy. Three replicates were measured for each condition, with at least 100 cells scored per replicate. Error bars = mean ± SD. p-values were calculated by one-way ANOVA followed by Tukey's multiple comparisons test.

(**C**) Coulter Counter-based cell volume measurements for RPE1 cells treated as indicated for 6 days. Each data point represents the mean cell volume obtained from five independent experiments. p-values were calculated by one-way ANOVA followed by Tukey's multiple comparisons test.

(**D**) Coulter Counter-based cell volume measurements for untreated (cycling), palbociclib-treated, and palbociclib + Torin1 treated MCF7 cells after 6 days.

(**E**) Data from (**D**) represented as a bar plot. Error bars = mean ± SD. p-values were calculated by one-way ANOVA followed by Tukey's multiple comparisons test.

(**F**) MCF7 cells were treated as in (**D**) and (**E**). After 6 days of treatment + 1 day of recovery in the presence of palbociclib, cells were seeded at ~250 cells/cm^2 in the absence of drugs for 10 days. Cells were then fixed and stained with crystal violet to visualize colonies.

(**G**) Quantification of (**F**). p-value was calculated by two-tailed, unpaired t-test. n=3. Error bars = mean ± SD.

(**H**) RPE1 WT cells were treated with the indicated doses of nutlin-3a (+/- Torin1) for 6 days. Cell sizes were measured by Counter Counter on Day 6. Error bars = mean ± SD. p-values were calculated by unpaired, two-tailed t-tests for the comparisons indicated.

(I) Colony formation assay for cells treated as in (**H**) + 1 day of recovery in nutlin-3a alone. Cells were then seeded at 250 cells/cm^2 in the absence of drugs for 8 days. Cells were then stained with crystal violet to visualize colonies.

(**J**) Western blots of whole cell lysates from a release time course following a 6-day G_1 arrest in size-constrained (Torin1-treated) and enlarged MCF7 cells. After 6 days, cells were washed into palbociclib-only media for 1 day before releasing into fresh media. Cell lysates collected at the indicated time points and probed with the indicated antibodies. GAPDH was used as a loading control.

(**K**) Representative Coulter Counter-based cell size measurements for the RPE1 FUCCI cells used in the experiments shown in **Figure 1H-1L**.

(**L**) Fractions of enlarged and size-constrained RPE1 WT cells (contact inhibition) that were binucleated or fragmented six days after G_1 arrest release. At this time, cells were fixed, nuclei were stained with Hoechst 33342, and nuclear defects were imaged by high content fluorescence microscopy. The fraction of fragmented nuclei and binucleated cells observed in each condition was calculated. Three replicates were measured for each condition, with at least 450 cells scored per replicate. Error bars = mean ± SD.

(**M**) NALM6 cells were treated with the indicated doses of palbociclib for 4 days, after which cells were fixed and DNA was stained with FxCycle FarRed. Cell cycle profiles were obtained by measuring FxCycle FarRed DNA stain incorporation by flow cytometry.

(**N**) NALM6 cells were treated with 1 μM palbociclib for 5 days, and cell volume was measured each day using a Coulter Counter. Note that cells were maintained at ~300,000 cells/mL for the duration of the experiment, though cell number only changed between Day 0 and Day 1. Day 0 indicates the time at which palbociclib was added.

(**O**) NALM6 cells were treated with the indicated concentrations of palbociclib (blue) for 6 days and were then released into EdU containing media in the absence of palbociclib for an additional 3 days. Cells were then fixed, and EdU was derivatized with AlexaFluor488 azide, followed by measurement of EdU incorporation by flow cytometry. The dashed line indicates EdU intensities that were considered EdU+ cells.

(**P**) NALM6 cells were treated with the indicated concentrations of palbociclib (blue) for 6 days. Cells were then spun down and re-diluted in drug-free media at a cell density of 100,000 cells/mL. Cells were then counted daily for 4 days following release. Error bars = mean ± SD for three replicates.

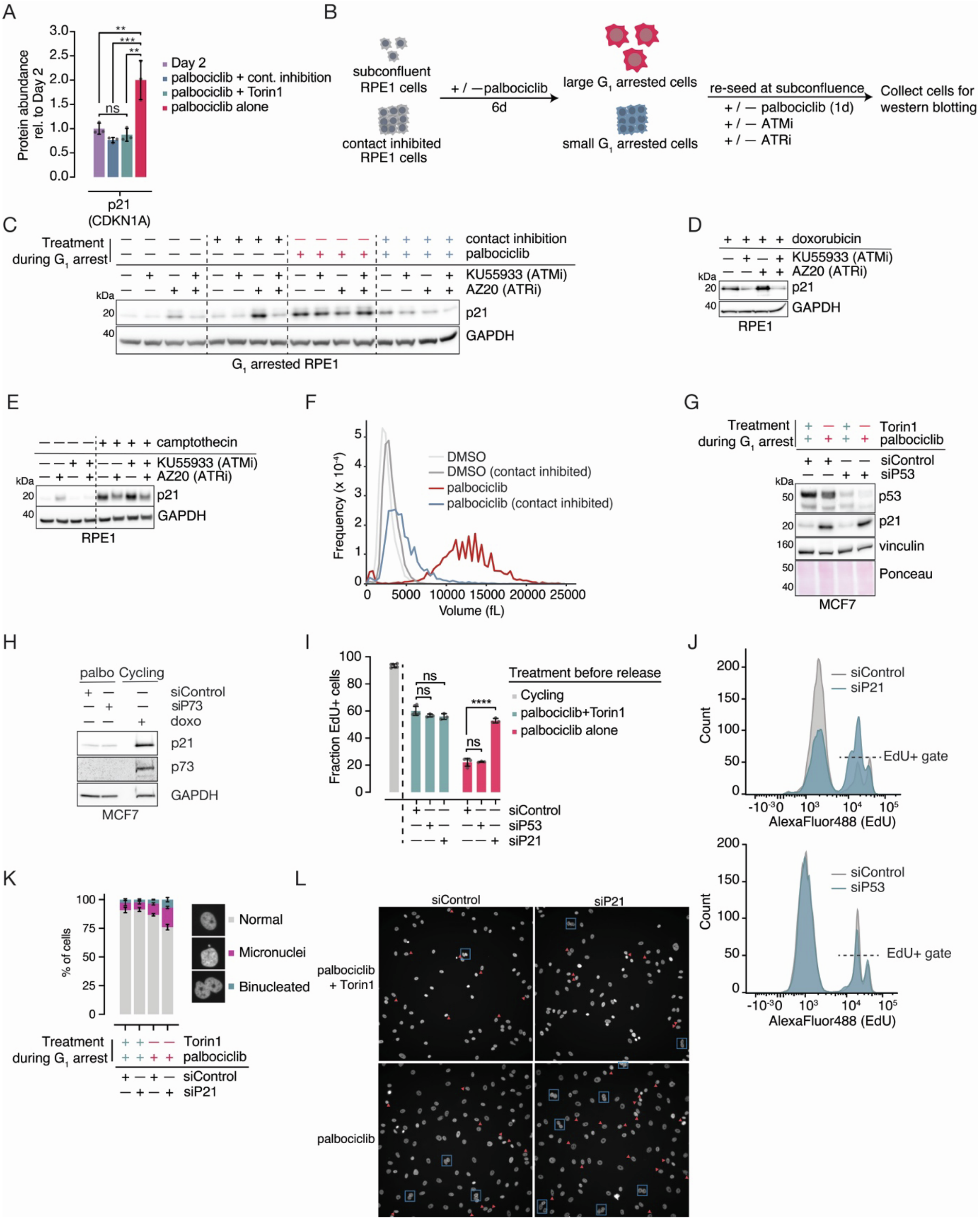

Figure S2: *Additional data related to Figure 2.*

(A) Quantification of p21 (CDKN1A) protein abundance based on MS measurements from the experiment shown in **Figure 3H**. Protein abundances were normalized to Day 2 samples. p-values were calculated by one-way ANOVA followed by Tukey's multiple comparisons test. Error bars = mean ± SD.

(B) Experimental scheme for measuring the effect of ATM and ATR signaling on p21 levels in enlarged and size-constrained G_1 arrested RPE1 cells. The small molecules KU55933 and AZ20 were used to block ATM and ATR activity, respectively.

(**C**) Experiment conducted using the scheme shown in (**B**). p21 levels were measured by western blotting, and GAPDH was used as a loading control.

(**D**) Validation that KU5933 can lower p21 levels as a function of ATM inhibition in RPE1 cells. Cycling RPE1 cells were treated with 1 μM doxorubicin +/- KU55933 and/or AZ20 for 24 hours before collecting. The indicated protein abundances were measured by western blotting. GAPDH was used as a loading control.

(**E**) Validation that AZ20 can lower p21 levels as a function of ATR inhibition in RPE1 cells. Cycling RPE1 cells were treated with DMSO or camptothecin +/- KU55933 and/or AZ20 for 24 hours before collecting. The indicated protein abundances were measured by western blotting. GAPDH was used as a loading control.

(**F**) Counter Counter based cell size measurements on Day 6 of the G_1 arrest (at the time of siRNA transfection) for the experiment shown in **Figure 2D-2I**.

(**G**) Enlarged and size-constrained MCF7 cells were treated as in **Figure 2B**. 24 hours after siRNA transfection in the presence of palbociclib, cells were collected, and the indicated protein abundances were measured by western blotting. Vinculin and Ponceau staining were used as loading controls.

(**H**) Enlarged MCF7 cells were treated as in (**G**), but instead of transfecting with a p53-directed siRNA were transfected with a p73-directed siRNA. 24 hours after siRNA transfection, cells were collected, and the indicated protein abundances were measured by western blotting. Cycling MCF7 cells treated with doxorubicin were analyzed in parallel as a positive control for the p73 antibody. GAPDH was used as a loading control.

(**I**) Size-constrained and enlarged MCF7 cells were treated with the indicated siRNAs for 24 hours in the continuous presence of palbociclib (as shown in **Figure 2B**) before releasing into EdU-containing drug-free media for three days. Cells were then collected, and EdU was derivatized with AlexaFluor488. EdU incorporation was then measured by flow cytometry. At least three replicates were analyzed for each condition. p-values were calculated by two-way ANOVA followed by Tukey's multiple comparison test. Error bars = mean ± SD.

(**J**) Examples of flow cytometry profiles obtained from the experiment described in (**I**). Dashed lines represent populations that were considered EdU+.

(**K**) Size-constrained (Torin1-treated) and enlarged MCF7 cells were treated as in (**I**) using a p21-directed siRNA for 24 hours in the presence of palbociclib prior to drug washout. Three days after release, cells were fixed, nuclei were stained with Hoechst 33342, and nuclear defects were imaged by high-content fluorescence microscopy. The fraction of micronuclei and binucleated cells observed in each condition were calculated. Four replicates were measured for each condition, with approximately

300 cells per replicate. For simplicity, cells that were binucleated and had micronuclei were only categorized as binucleated. Error bars = mean ± SD.

(**L**) Representative images for the experiment described in (**K**). Binucleated cells are indicated by blue boxes, and micronuclei are denoted with red arrows.

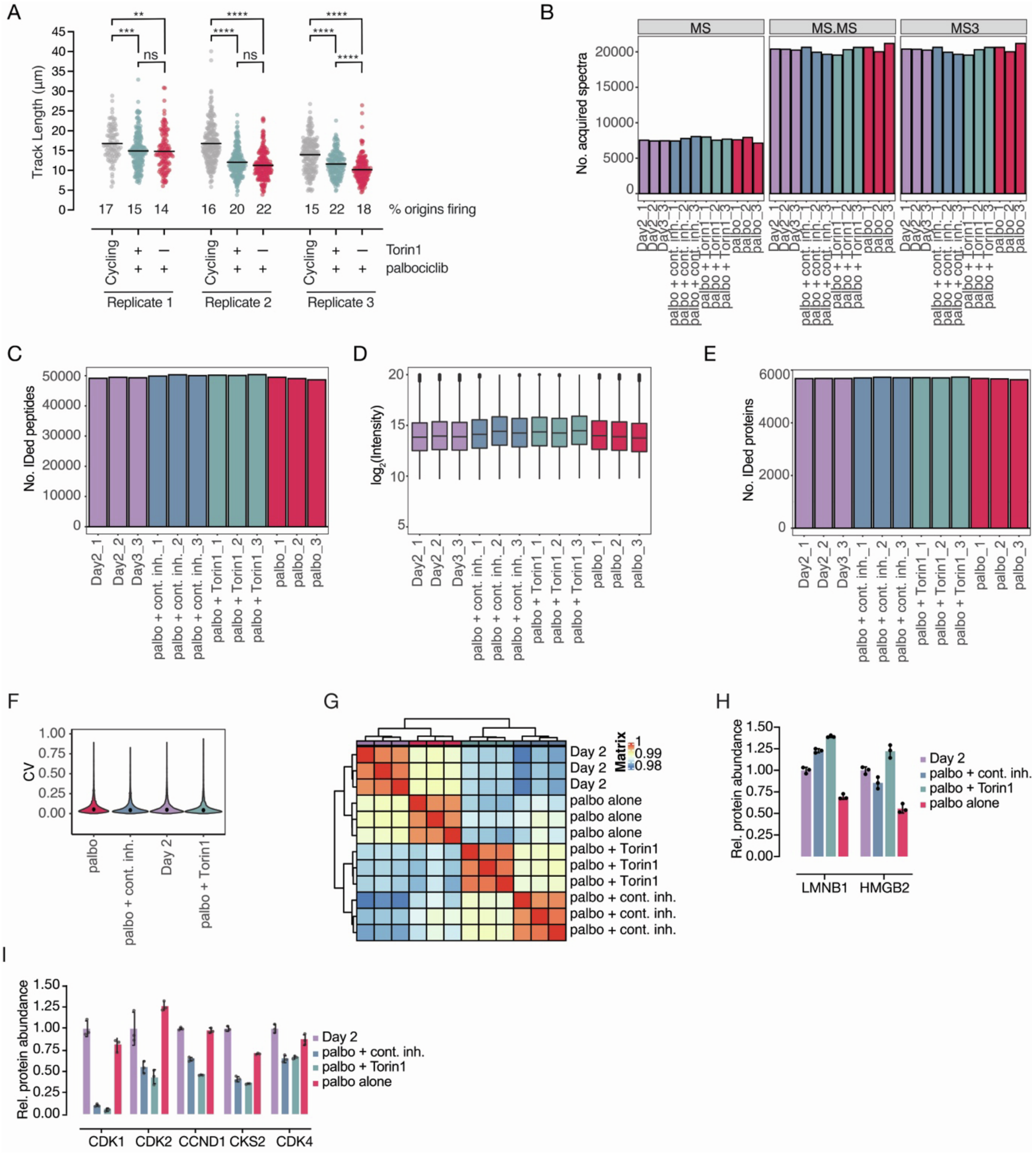

Figure S3: *Additional data related to Figure 3.*

(A) Data from **Figure 3B** re-plotted with replicates separated. Bars represent the median track length for each condition. p-values were calculated by Kruskal-Wallis test (one-way ANOVA on ranks).

(B) Number of spectra acquired in MS1, MS2, and MS3 for each reporter ion channel per acquired run for the G_1 cell size TMT proteomics experiment described in **Figure 3**.

(**C**) Number of peptides identified per reporter ion channel.

(**D**) Boxplot of peptide intensities per reporter ion channel.

(**E**) Number of proteins identified per reporter ion channel.

(**F**) Coefficient of variation for protein intensities in each condition.

(**G**) Unsupervised hierarchical clustering analysis of the replicates for each condition.

(**H-I**) Protein abundances of known senescence markers (**H**) and various positive regulators of the G_1/S transition (**I**) as measured by mass spectrometry. Protein abundances were normalized to the Day 2 samples.

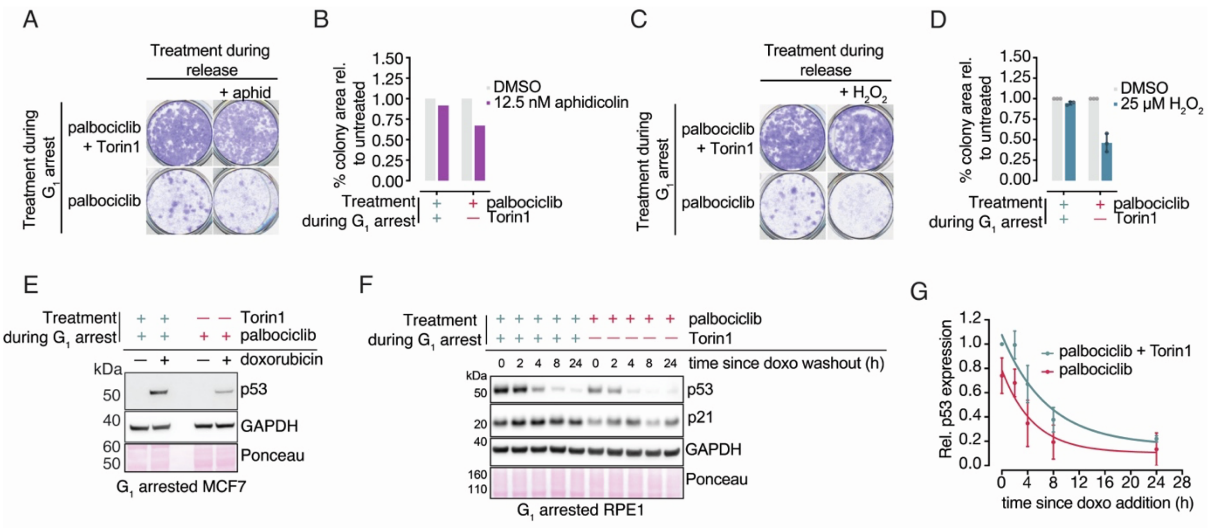

Figure S4: *Additional data related to Figure 4.*

(**A**) RPE1 cells were treated as in **Figure 1A** and were re-seeded at ~250 cells/cm^2 in DMSO-containing media or 12.5 nM aphidicolin. Cells were then fixed and stained with crystal violet to visualize colonies after 10 days.

(**B**) Quantification of (**A**). n = 1. Samples were normalized to the DMSO treatment condition for each cell size condition.

(**C**) RPE1 cells were treated as in **Figure 1A** and were re-seeded at ~250 cells/cm^2 in normal media or 25 µM hydrogen peroxide. Cells were then fixed and stained with crystal violet to visualize colonies after 10 days.

(**D**) Quantification of (**C**). n = 3. Samples were normalized to the untreated condition for each cell size condition.

(E) MCF7 cells were treated as in **Figure 4A** with 500 nM doxorubicin. Cells were collected after 24 hours, and the indicated protein abundances were measured by western blot. GAPDH and Ponceau staining were used as loading controls.

(**F**) RPE1 cells were treated as in **Figure 4A** with 500 nM doxorubicin in the presence of palbociclib for 16 hours before washing the doxorubicin out (maintaining the palbociclib) and taking samples for western blotting at the indicated time points. Protein abundances were measured by western blot using the indicated antibodies. GAPDH and Ponceau staining were used as loading controls.

(**G**) Quantification of p53 levels from the experiment shown in (**F**). Protein levels were normalized to GAPDH intensity and then to the 0 hr size-constrained time point. Error bars = mean ± range for two experiments.

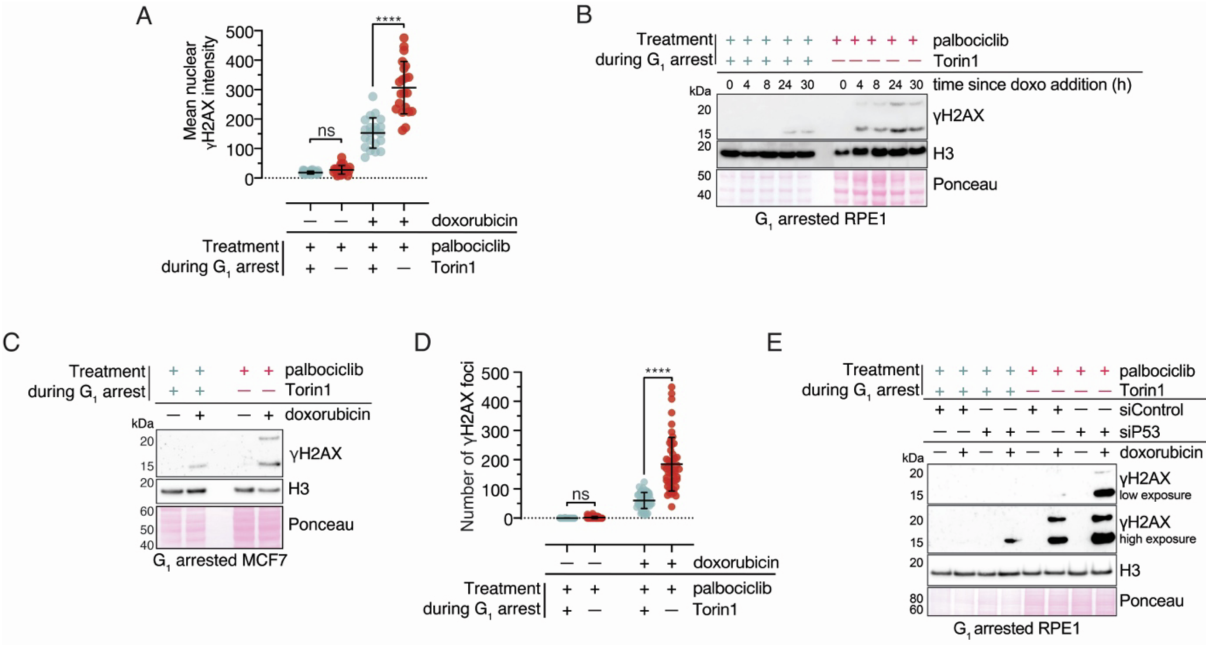

Figure S5: *Additional data related to Figure 5.*

(A) Re-analysis of the experiment in **Figure 5A-5B**, where γH2AX is quantified using mean nuclear intensity rather than foci count.

(**B**) Enlarged and size-constrained G_1 arrested RPE1 cells were treated with 1 μM doxorubicin in the continuous presence of palbociclib. Cells were collected at the indicated time points, and the indicated protein abundances were measured by western blot. Loading was normalized to cell number rather than protein abundance to account for histone sub-scaling. Histone H3 was used as a loading control, and Ponceau staining was used to illustrate differences in total protein content.

(**C**) Enlarged and size-constrained MCF7 cells were treated as in (**B**) with 500 nM doxorubicin and were collected after 24 hours. Loading was normalized to cell number, and the indicated protein abundances were measured by western blot. Histone H3 was used as a loading control, and Ponceau staining was used to illustrate differences in total protein content.

(**D**) MCF7 cells were treated as in (**C**) and γH2AX foci were analyzed by immunofluorescence. 50 cells were analyzed for each condition. p-values were calculated by two-way ANOVA followed by Tukey's multiple comparisons test.

(**E**) Enlarged and size-constrained cells were treated as in **Figure 5F** but were collected for western blotting against the indicated proteins. Loading was normalized to cell number. Histone H3 was used as a loading control, and Ponceau staining was used to illustrate differences in total protein content.

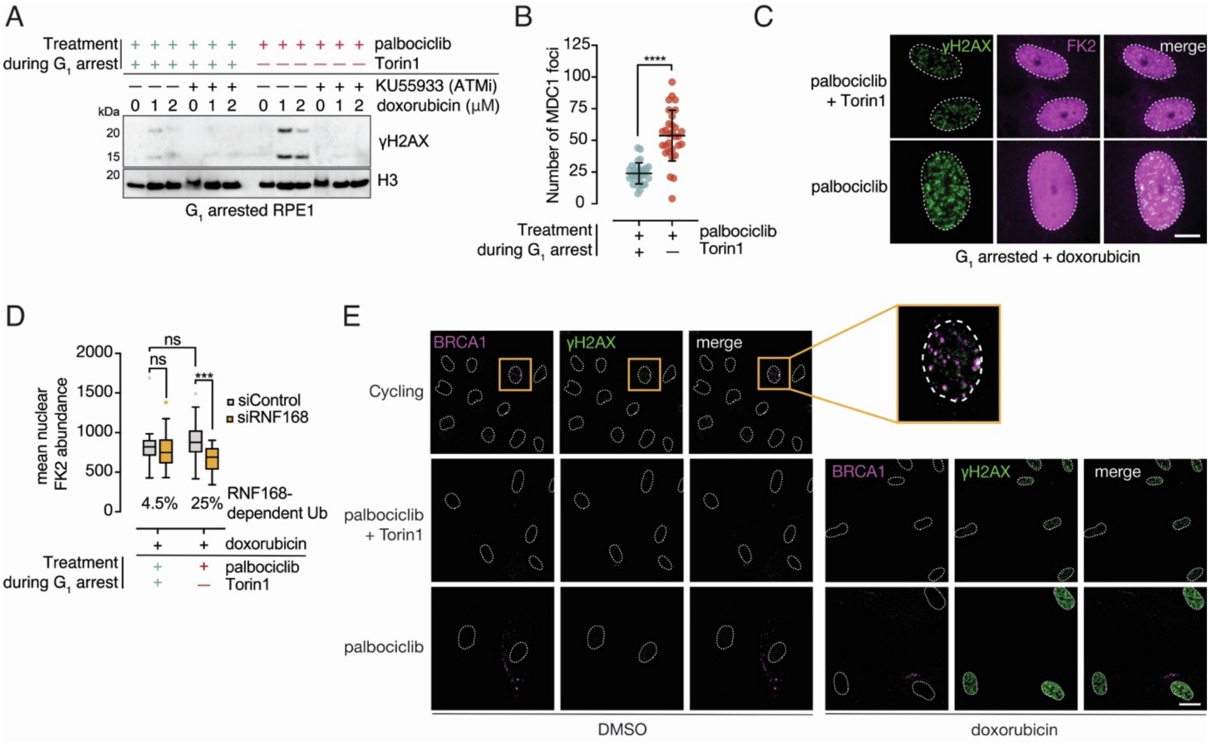

Figure S6: *Additional data related to Figure 6.*

(**A**) G_1 arrested enlarged and size-constrained RPE1 cells were treated +/- KU55933 (an ATM inhibitor) and the indicated doses of doxorubicin in the continuous presence of palbociclib. Cells were collected after 24 hours and lysed. Loading was normalized to cell count rather than protein concentration to account for histone sub-scaling. The indicated protein abundances were measured by western blotting. Histone H3 serves as a loading control for histone abundance.

(**B**) Quantification of the MDC1 foci from the experiment described in **Figure 6D-6E**.

(**C**) G_1 arrested enlarged and size-constrained cells were treated with doxorubicin for 24 hours in the continuous presence of palbociclib. Cells were then immunostained for γH2AX and FK2, a polyubiquitin antibody. Hoechst 33342 was used as a nuclear counterstain (white dashed outline). Scale bar = 10 μm.

(**D**) Mean nuclear ubiquitin measurements for the experiment described in (**C**). Mean FK2 fluorescence was measured for individual nuclei and background subtracted. At least 30 cells were quantified per condition. Data are represented as Tukey plots. Percentage of RNF168-dependent ubiquitin was calculated as: $[mean_{siControl} - mean_{siRNF168})/mean_{siControl}] \times 100$. p-values were calculated by two-way ANOVA followed by Tukey's multiple comparisons test.

(**E**) Representative images for the experiment described in **Figure 6I**. Scale bar = 25 μm.
